# PEGylated Recombinant Human Growth Hormone Jintrolong^®^ Exhibits Good Long-Term Safety in Cynomolgus Monkeys and Human Pediatric Growth Hormone Deficiency Patients

**DOI:** 10.3389/fendo.2022.821588

**Published:** 2022-07-15

**Authors:** Wei Wu, Juan Zhou, Chuandong Wu, Qian Zhou, Xiaoyu Li, Yanlin Zhang, Conglin Zuo, Jun Yin, Ling Hou, Shuyang Wang, Hongyang Gao, Tianhong Luo, Lei Jin, Enhong Zhong, Yingwu Wang, Xiaoping Luo

**Affiliations:** ^1^Department of Pediatrics, Tongji Hospital, Tongji Medical College, Huazhong University of Science and Technology, Wuhan, China; ^2^Center for Nonclinical Research and Translational Medicine, Changchun GeneScience Pharmaceuticals Co., Ltd., Changchun, China; ^3^Department of Toxicology, JOINN Laboratories (Suzhou) Co., Ltd., Suzhou, China; ^4^Department of Pathology, School of Basic Medical Sciences, Fudan University, Shanghai, China; ^5^Electron Microscope Core Laboratory, Shanghai Medical College, Fudan University, Shanghai, China; ^6^School of Life Science, Jilin University, Changchun, China

**Keywords:** PEG-rhGH, GHD, choroid plexus (CP), vacuolation, MRI, TEM, CNS, AE

## Abstract

Jintrolong^®^ is a long-acting PEGylated recombinant human growth hormone (PEG-rhGH) developed for weekly injection in patients with pediatric growth hormone deficiency (PGHD). Although PEG modification of therapeutic proteins is generally considered safe, concerns persist about the potential for adverse vacuolation in tissues with long-term exposure to PEG-included therapies, particularly in children. We assessed the safety of Jintrolong^®^ in cynomolgus monkeys with an examination of vacuolation in the brain choroid plexus (CP) and reported long-term clinical safety data obtained from children with PGHD. The toxicity of Jintrolong^®^ was assessed following the 52-week administration with doses at 0.3, 1, or 3 mg/kg/week. The levels of vacuolation of CP in animals were dose-dependent and at least partially reversible after a 104- or 157-week recovery period. Vacuolation in the CP epithelium did not lead to obvious subcellular structural or cell functional abnormalities. Compared with the clinical dose of 0.2 mg/kg/week Jintrolong^®^ in PGHD patients, exposure in monkeys under NOAEL 3 mg/kg/week exhibited safety margins greater than 120.5, the predicted minimum dose to induce vacuolation in monkeys is equivalent to 1.29 mg/kg/week in humans, which is 6.45-fold higher than the clinical dose. The safety data acquired in clinical trials for Jintrolong^®^ were also analyzed, which included phase III (360 patients), phase IV (3,000 patients) of 26-week treatment, and a follow-up study with treatment lasting for 3 years. There was no statistically significant difference in the incidence of adverse reactions between the Jintrolong^®^ group and the daily rhGH control group (no PEG), and no new adverse effects (AE) were observed in the Jintrolong^®^ group at the clinical therapeutic dose of 0.2 mg/kg/week.

## Introduction

Growth hormone deficiency (GHD) is a condition caused by insufficient amounts of growth hormone in children and adults (1–3). The incidence of GHD has been estimated to be about 1 in 4,000 to 1 in 30,000 in children worldwide (4, 5), while the incidence of adults with profound GH deficiency has been reported with an estimated prevalence of 350 out of 1,000,000 (4, 5). Recombinant human GH is an important pharmacologic agent used to stimulate growth in children with GHD (1).

Pediatric GHD patients receive years of daily rhGH subcutaneous (SC) injections since the serum half-life of rhGH is only 3.4 h (6, 7). Approximately 23% of teenage patients miss two or more injections per week because daily injections are distressing and inconvenient, resulting in poor compliance and treatment outcomes (8). To improve patient compliance, several long-acting rhGH preparations have been developed recently. Nutropin Depot, the first long-acting growth hormone (LAGH) formulation of GH-capsulated microspheres, was removed from the market because of its high manufacturing cost (9). In addition to the microsphere formulation of LAGH, other protein-fusion LAGH formulations such as albumin-fused JR-142 (10), FC-fused GX-H9, and VRS-317 (11, 12) are still in development. The XTEN-fused VRS-317 related Phase 3 study in pediatric GHD has been terminated as the primary endpoint of non-inferiority to daily therapy was not achieved. Albumin-binding Sogroya^®^ with an acyl linker was approved by the FDA in August 2020 for use in adults with GHD but has yet to be marketed (13). Carboxy-terminal peptide of human chorionic gonadotropin (hCG)-fused MOD4023 (14) has just been approved by Health Canada in October 2021 as NGENLA but lacks long-term clinical safety data.

One strategy to improve the pharmacokinetic (PK) characteristics of LAGH is to add a non-antigenic polyethylene glycol (PEG) moiety, which prolongs the serum half-life of GH by decreasing its clearance *via* the kidneys. PEGylated LAGH formulations have been developed (15), with examples such as PHA-794428, NNC126-0083, ARX201, BBT-031, and Jintrolong^®^ (16). However, some of these PEGylated LAGH drugs have faced significant setbacks and challenges. The development of PHA-794428 was terminated because of a high rate of lipoatrophy at the injection site, and PHA-794428 was not developed further (17). NNC126-0083 was stopped for its unsatisfactory IGF-I profile peak and duration (18). ARX201 is no longer developed due to observation of vacuolation in the choroid plexus (CP) of monkeys (9). BBT-031 is still under evaluation in preclinical studies (10). TransCon rhGH Skytrofa^®^ (19) was just approved for market entry by the FDA in August 2021 for use in pediatric GHD but lacks long-term clinical safety data. Jintrolong^®^ (PEG-rhGH), containing a 40 kDa branched PEG attached to the amino group of rhGH, was approved by the National Medical Products Administration (NMPA) of China in 2014 (20) as the first PEGylated rhGH formulation for pediatric patients with GHD. Jintrolong^®^ demonstrated comparable serum IGF-1 levels compared to daily GH yet slower clearance and treatment with Jintrolong^®^ produced no severe adverse events during a Phase I trial (7). A phase II trial established the preliminary efficacy, and safety and recommended Jintrolong^®^ dose at 0.2 mg/kg/week. A phase III trial demonstrated a greater height growth velocity increase in patients treated weekly with Jintrolong^®^ versus daily GH among children with GHD (21). A 24-month follow-up study demonstrated that Jintrolong^®^ is effective, well-tolerated, and convenient to administer in Chinese children with GHD (8). Additionally, the safety, tolerability, and pharmacokinetics of Jintrolong^®^ in adult subjects were also evaluated to be well tolerated (22).

Although the PEGylation of drugs is generally considered safe (23–25), concerns persist about the potential adverse effects of long-term exposure, especially considering that children with GHD may need PEG-rhGH treatment for years. The major potentially adverse effect ascribed to PEG in non-clinical toxicology studies is cellular vacuolation (23). Cellular vacuolation is a frequently observed histopathological phenomenon upon exposure to bacteria, viral pathogens, or pharmaceutical agents in which cells develop vacuoles or vacuole-like structures in lysosomes, autolysosomes, endosomes, and endoplasmic reticulum. These vacuoles may grow in size and prevalence with increased exposure (15, 26–30).

There is limited information about the role of vacuolation in cytotoxicity (31). Cytoplasmic vacuolation is considered a normal physiological adaptive response to remove foreign materials and protect the cell against toxins (27). However, prolonged exposure to drugs and resultant cellular vacuolation could induce irreversible cellular injuries by compromising the cytoarchitecture of affected cells and/or tissues (29, 32, 33). Since PEGylated drugs do not undergo normal renal clearance when the molecular weight of PEG is more than 30 kDa, they are more persistent in the circulation, so typical tissues and cell types affected by PEG-induced vacuolation include those with rich blood flow and perfusion, such as renal tubule cells, circulating macrophages, and the CP (24). The kidney appears to be the primary target organ for the histological effects of high molecular weight PEGs (28), yet several publications reported no adverse effects on kidney function despite significant cytoplasmic vacuolation of renal tubular epithelium cells (15, 28, 31). PEG-induced vacuolation in macrophages is deemed a normal result of the function of macrophages in removing foreign materials and can be partially or completely recovered after a treatment-free period (15, 34). The PEG-induced vacuolation in the CP, however, is of concern in previous preclinical safety assessments of PEGylated drugs (35–37). The CP comprises epithelial cells with tight junctions and fulfills the important function of producing and regulating the amount of cerebral spinal fluid (CSF) present in the brain (38, 39). In previous reports, the vacuolation of epithelial cells in CP was not readily reversible because these epithelial cells are slowly renewable cells (15). The Pediatric Committee (PDCO) of the European Medicines Agency (EMA) summarized several cases of observed cellular vacuolation caused by PEGylated drugs in non-clinical studies and listed the conditions under which to observe PEG-related vacuolation, including: 1) drugs where the PEG molecular weight exceeds 40 kDa; 2) the PEGylated LAGH exposure exceeds 0.4 μmol/kg/month; 3) the drugs were tested in cynomolgus monkeys; 4) the duration of administration is at least 6 weeks (26). However, the PDCO did not define cellular vacuolation or its effects as adverse findings or recommend an appropriate safety margin for PEGylated LAGH. The Growth Hormone Research Society agreed that the safety of longer exposure to LAGH is still unclear (9).

The LAGH drug Jintrolong^®^, containing a 40 kDa PEG moiety, was approved for clinical use in 2014 at a dose of 0.2 mg/kg/week, and its safety has been widely reviewed by several institutions (5, 9, 10, 16, 20). The dose of 0.2 mg/kg/week is equal to 0.009 µmol PEG/kg/week, which is about 11-fold lower than the concentration of 0.4 μmol PEG/kg/month cited in the summarization of PDCO (26). Considering that children with GHD may require Jintrolong^®^ treatment for more than 5 years, the toxicity of Jintrolong^®^ over long-term administration was further assessed to determine whether long-term repeated Jintrolong^®^ administrations induce cellular vacuolation in tissues such as the CP, whether vacuolation is dose-dependent and reversible, and whether PEG-induced vacuolation in the CP significantly affects subcellular structure, glucose metabolism, or neurobehavioral indices. To further evaluate the clinical safety of Jintrolong^®^, especially for possible CP-related adverse reactions, pediatric GHD Phase III (360 patients) and Phase IV (3,000 patients) studies with 26 weeks of treatment were analyzed, and a follow-up study of Phase IV with treatment time as long as 3 years was also conducted, comparing the incidence of adverse events following long-term Jintrolong^®^ or daily rhGH treatment. The combination of nonclinical and clinical studies described in this report details the comprehensive safety profile of Jintrolong^®^.

## Materials and Methods

### Test and Control Articles

Jintrolong^®^, provided by GeneScience Pharmaceuticals (Changchun, China), is a PEGylated rhGH in which the rhGH molecule is covalently attached *via* its amino terminus to a 40 kDa branched hydrophilic PEG residue. The molecular weight of Jintrolong^®^ is around 62 kDa, with 40 kDa PEG, and 22 kDa rhGH. When Jintrolong^®^ was applied in nonclinical and clinical studies, the dose was calculated based on hGH. For example, the clinical dose 0.2 mg/kg/week means 0.2 mg hGH/kg/week of Jintrolong^®^ (corresponding to 0.009 µmol/kg/week).

The daily rhGH Jintropin, provided by GeneScience Pharmaceuticals, is rhGH without any PEGylation and was used as a control in the clinical trials. Injection Blank Excipient Solution, which includes sodium citrate, phenol, poloxamer 188, and sodium chloride, was used as the vehicle control without PEG or rhGH in the preclinical safety assessment study.

### Animals

To fully assess the preclinical toxicity of Jintrolong^®^, non-human primate cynomolgus monkeys were chosen as a standard animal model for toxicological research and testing with a large amount of background data. Sixty-four cynomolgus monkeys (32/sex) were supplied by the Guangxi Guidong Non-human Primate Development and Experiment Ltd (Guangxi, China). The age of cynomolgus monkeys from 12 to 24 months corresponds to an age of 4–8 years old in human children, and thus monkeys in this age range were selected for this study. Animals were housed in an environmentally monitored, well-ventilated conventional-grade room. Fruits and certified non-human primate diets (Beijing Keao Xieli Feed Co. Ltd, SCXK (Jing) 2014-0010 and SCXK (Jing) 2019-0003) were provided daily except for scheduled fasting periods. Water was provided *ad libitum* during the study period. Animal care complied with the standard operating procedures (SOPs) of JOINN Laboratories (Suzhou). JOINN Laboratories (Suzhou) is fully accredited by the Association for Assessment and Accreditation of Laboratory Animal Care International (AAALAC). The non-clinical laboratory study conducted at JOINN Laboratories (Suzhou) was compliant with the current U.S. Food and Drug Administration (FDA) current Good Laboratory Practice (GLP) regulations (21 CFR Part 58), OECD Principles of Good Laboratory Practice (as revised in 1997), ENV/MC/CHEM (98) 17, Former China FDA GLP regulations (CFDA, Decree No. 34, 2017), and the Standard Operating Procedures (SOPs) of JOINN Laboratories (Suzhou).

### Randomization and Group Assignment

All monkeys were randomly assigned to designed treatment groups by using a computer-generated randomization procedure to make groups comparable in body weight. The grouping and dosing information were listed in **Table 1**. Randomization was performed on Day −2 (two days prior to first drug administration).

**Table 1:** Animal grouping and test article dosing information.

Group	Drug administered	rhGH Dose Level (mg/kg/week)^d^	PEGContent(μmol/kg/week)	DoseVolume(ml/kg)	Total number of animals	Number of animals for 26-week terminal necropsy	Number of animals for 52-week terminal necropsy	Number of animals for 104-week terminal necropsy	Number of animals for 157-week terminal necropsy
1	Vehicle control	0	0	0.33	18	6	8	2	2
2	Jintrolong^®^	0.3	0.0135	0.033	12	0	8	2	2
3		1	0.045	0.11	10	6	4	0	0
4		1	0.045	0.11	12	0	8	2	2
5		3	0.135	0.33	12	0	8	2	2

Each monkey received the test article or excipient control *via* subcutaneous injection using a 1 ml aseptic syringe on the hind limb. The dosage is accurate to 2 decimal places. The dose of each animal was adjusted according to the most recently measured body weight. Animals were euthanized using ketamine (10 mg/kg, 50 mg/ml) *via* intramuscular injection and sodium pentobarbital solution (20 mg/kg, 20 mg/ml) *via* intravenous injection, or directly euthanized using Zoletil 50 (12 mg/kg, 50 mg/ml) *via* intramuscular injection followed by femoral artery exsanguination in accordance with the AVMA Guidelines for the Euthanasia of Animals: 2013 Edition (the American Veterinary Medical Association, 2013).

### Tissue Slide Preparation and Histopathological Examination

Tissues such as the brain CP, kidney, lung, liver, heart, and spleen were collected from all animals following necropsy examinations and processed using routine histological methods at JOINN Laboratories (Beijing), where tissues were embedded in paraffin, sectioned, mounted on slides, and stained with hematoxylin and eosin (H&E). The maximum width of brain coronal sections containing bilateral ventricles was stained to fully observe and characterize the cytoarchitecture of the CP. Findings on the slides were scored and categorized using a standardized nomenclature. A four-step grading system of minimal, mild, moderate, or severe was used to grade the severity of microscopic lesions for comparison among groups. Pathology assessments were double-blind; the pathologists were given only monkey numbers without any monkey grouping information.

### Immunohistochemistry for Anti-PEG and Ki-67

Paraffin-embedded brain tissue sections from groups 1 (control group) and 5 (3 mg/kg/week Jintrolong^®^ group) were evaluated using an immunohistochemistry method for anti-PEG and markers of cell proliferation (Ki-67). The paraffinized brain sections from all euthanized animals were shipped to West China-Frontier PharmaTech Co., Ltd. at room temperature and evaluated using anti-PEG and anti-Ki-67 staining. Deparaffinized sections were rehydrated, quenched in 3% hydrogen peroxide for 20 min at 10–30°C, blocked by normal goat serum working fluid, followed by incubating with primary antibody 1:25 anti-PEG (PEG-B-47, Abcam) or 1:250 anti-Ki-67 (ZM-0166, ZSGB-BIO) overnight. After incubation with 1:250 peroxidase-conjugated goat anti-rabbit IgG, sections were developed with a DAB peroxidase substrate kit (DAB-0031/1031) according to manufacturer instructions. The slides were counterstained with hematoxylin, dehydrated, and mounted.

### Transmission Electron Microscopy Examination

Brain tissue samples containing the CP were collected from all animals at the terminal necropsy after the last dosing and fixed with 2.5% glutaraldehyde in 0.1 mol/L phosphate buffer (pH 7.2) for hours. The samples were sealed in vials and stored at 2–8°C before being shipped to the Shanghai Medical College of Fudan University for further processing. The fixed samples were rinsed in the phosphate buffer. The post-fixation period was observed for 2 h with 1% osmium tetroxide at 4°C, after which the samples were dehydrated and embedded in E-51 epoxy resin (Shanghai Resin Factory Co., Ltd., China). Ultrathin sections were prepared using a Leica ultramicrotome (Leica Microsystems Ltd., Germany), and counter staining with uranyl acetate and lead citrate was performed. The ultrastructure of the CP was examined using a Philips CM120 electron microscope (FEI Company, USA) at 60 kV and a Gatan camera.

### Functional Observational Battery for Neurobehavioral Assessments

Functional observational battery (FOB) measurements for neurobehavioral assessments (home cage observations, out-of-cage observations, and open field activity assessments) were designed to be compliant with the goals of standard veterinary neurological examinations and federal and international regulatory guidelines related to neurotoxicity safety assessments (40). FOB measurements were conducted in the vehicle control group and Jintrolong^®^-treated groups before euthanasia following the last dosing (Day 368), the 53 weeks of the recovery period (Day 739), and the 104 weeks of the recovery period (Day 1095). The observations were conducted by two independent single-blinded observers on all occasions.

### Magnetic Resonance Imaging (MRI)

The first 2 animals/sex in Group 1 (control group) and group 5 (3mg/kg/week Jintrolong^®^ group) at the terminal points after the last dosing were sent to the Nanjing Medical University for brain magnetic resonance imaging (MRI) under non-GLP conditions. Before the examination, animals were anesthetized using 10 mg/kg of ketamine *via* intramuscular injection, followed by 20 mg/ml of 0.2 µm filtered sodium pentobarbital solution *via* intravenous injection within a dose range of 5–20 mg/kg.

### Positron Emission Tomography (PET) With Fluorine-18 Fluorodeoxyglucose

All animals were transferred to Huayi Molecular Imaging Institute on Day 1,403, and positron emission tomography (PET) using fluorine-18 fluorodeoxyglucose was conducted to evaluate the glucose metabolism function of the brain under non-GLP conditions. Before the examination, animals received Zoletil 50 (4–12 mg/kg, 50 mg/ml) *via* intramuscular injection, and then received 18F-FDG (1 mCi/kg, 1 ml/animal) *via* intravenous injection. Animals were in the prone position for approximately 60 min and scanned for approximately 10 min. All animals were transferred to the No. 1 People’s Hospital of Changshu on Day 1,463, and an MRI examination was conducted on the brain to define the location of each brain tissue of each animal for the 18F-FDG-PET data analysis under non-GLP conditions. Before the examination, animals first received Zoletil 50 (4–12 mg/kg, 50 mg/ml) *via* intramuscular injection, and then were scanned for approximately 15 min.

### Cerebrospinal Fluid Analysis

Approximately 1 ml of cerebrospinal fluid (CSF) samples were collected from all animals with a syringe *via* a foramen magnum puncture before the scheduled necropsy for each animal. The color of cerebrospinal fluid samples was observed and recorded, followed by a count of white blood cells (WBC) and red blood cells (RBC) measured *via* a hemocytometer. A TBA-120FR automatic biochemical analyzer was used to determine the total protein content, albumin, and globulin in the CSF samples. The ratio of these proteins to serum and CSF was calculated and compared.

### Toxicokinetic Parameters in Cynomolgus Monkeys

Blood samples of approximately 1 ml were collected from all animals *via* the forelimb or hindlimb vein for toxicokinetic (TK) analysis. Samples of whole blood were added to centrifuge tubes without anticoagulant and stored on ice for serum separation. The blood samples were centrifuged at 1,500×*g* for 10 min at 4°C within 2 h after collection. After the centrifugation, the serum was aliquoted into three new tubes and stored below −18°C. One set of serum samples was used to analyze the concentration of IGF-1 (insulin-like growth factor-1), and all other serum samples were used for TK analysis. TK parameters such as the area under the curve (AUC) and maximum plasma concentration (C_max_) were calculated using WinNonlin software using non-compartmental analysis. Microsoft Office Excel was used for data statistical analysis, including mean, standard deviation (SD), and coefficient of variation (CV%). Differences in TK parameters between males and females were compared.

### Quantification of Vacuolation in the Cynomolgus Monkeys

All H&E stained monkey brain sections were scanned with a digital pathology slide scanner and analyzed with NDP view 1.2.46 software. Based on the Halo AI artificial intelligence tissue typing system, the whole tissue area was circled, and the blank area and staining artifacts (folds in the tissue and non-specific staining) were removed. The epithelial tissue and non-epithelial areas are labeled, respectively, based on the HALO AI DenseNet convolutional neural network algorithm. A total of 52,400 iterations of training were performed with an image resolution of 2 μm/pixel (×40), achieving a Cross-Entropy score of less than 0.1. The area of the epithelial tissue area and its percentage of the analysis area were identified and calculated. In the identified epithelial tissue region, the vacuolar and non-vacuolar tissue regions were labeled, respectively, to establish the algorithm model based on the Halo AI densenet convolution neural network algorithm. A total of 10,000 iterative training sessions are carried out with an image resolution of 0.22 μm/pixel (×40) till the Cross-Entropy is less than 0.1. The total area of vacuoles, the number of vacuoles, the perimeter, and the area of each vacuole were identified and quantified. In the identified epithelial tissue region, the nucleus and other regions were labeled, respectively, to establish the algorithm model. A total of 51,000 iterative trainings were performed with an image resolution of 0.25 μm/pixel (×35.2) until the cross entropy was less than 0.1. The total number of cells in the epithelial area, the area of the total cell area, the perimeter, and area of each cell were calculated. A pathologist analyzed and compared semi-quantification data with representative quantification data. The algorithm was applied to each tissue slice for objective quantitative analysis after the algorithm debugging.

### Model-Based Prediction of Vacuolation Formation and Recovery in the Cynomolgus Monkeys and in Clinical Pediatric GHD

Due to the administration time and dose difference between cynomolgus monkeys and humans, we must generate a pharmacokinetic (PK) model to bridge the preclinical and clinical studies. Serial blood samples were collected from animals up to 8,736 h postdose for evaluating the PK of PEG-rhGH. The PK data of PEG-rhGH in cynomolgus monkeys were modeled using the pharmacokinetic modeling software package NONMEM (v7.4, ICON Development Solutions, Ellicott City, Maryland, USA).

The PK model structure in the cynomolgus monkey is illustrated below:

           
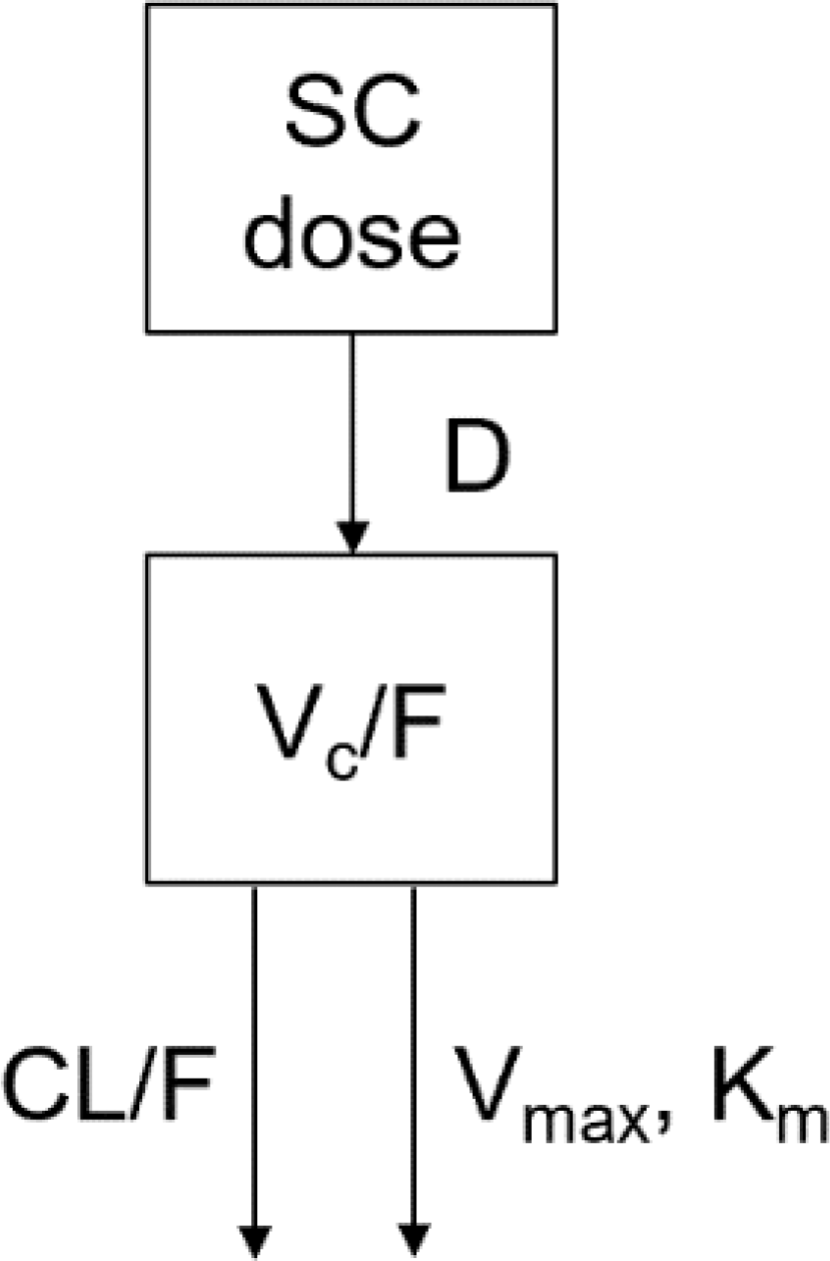


A one-compartment model with zero-order absorption and parallel first-order and nonlinear elimination pathways from the central compartment was developed to describe the PK of PEG-rhGH in cynomolgus monkeys.

Serum concentrations of PEG-rhGH in the center are symbolized by C. CL represents the clearance, and Q is the inter-compartmental clearance between the central compartment and peripheral compartment. Km is the Michaelis constant, and Vmax is the maximum rate of elimination of the nonlinear pathway. Vc is the volume of distribution of the central compartment. F stands for bioavailability.

Assume that the inter-individual variability (IIV) is log-normal distributed:


$$\theta_{i}=\theta_{TV}\cdot e^{\eta_{i}}$$

θ*i*; is the parameter of the ith individual, *θ_TV_* is the typical value of the population, and *η_i_* is the random variable that stands for inter-individual variability and follows the normal distribution with the variance of ω2.

The residual is explained by the proportional error and the additional error model:


$$Y_{ij}=\;C_{ij}\;\cdot \left(1+\epsilon_{ij}\right)+\xi_{ij}$$

θj is the serum concentration observed in the ith individual at time j, θj is the predicted concentration, ϵ is the proportional error, and ξ is the additional error. Assume that the ϵ and ξ are normally distributed with the mean of zero and the variance of $\sigma_{\epsilon }^{2}$ and $\sigma_{\xi }^{2}$ .

The PD model structure in the cynomolgus monkey is illustrated below:


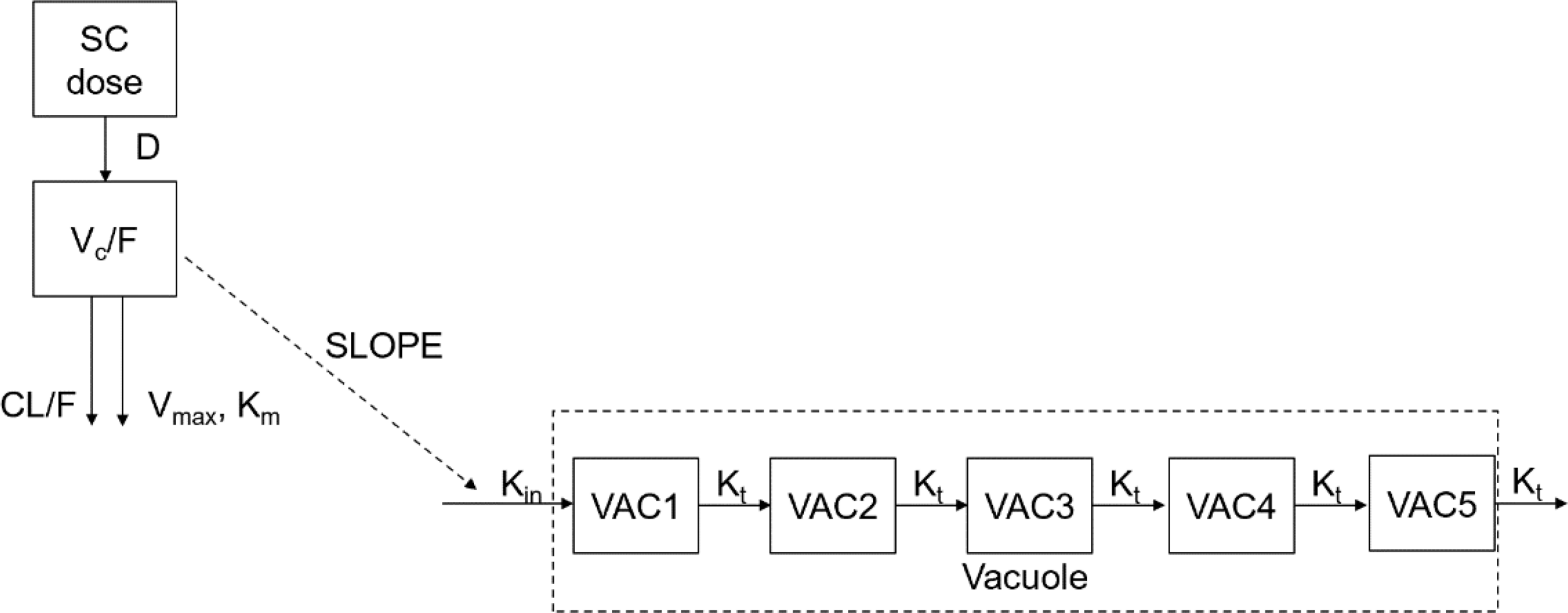


A transit-compartment PD model was developed to describe the vacuolation (vacuole/cell number) of cynomolgus monkeys after SC administration of PEG-rhGH.

The PK data of PEG-rhGH in pediatric patients were modeled using the model structure below:

          
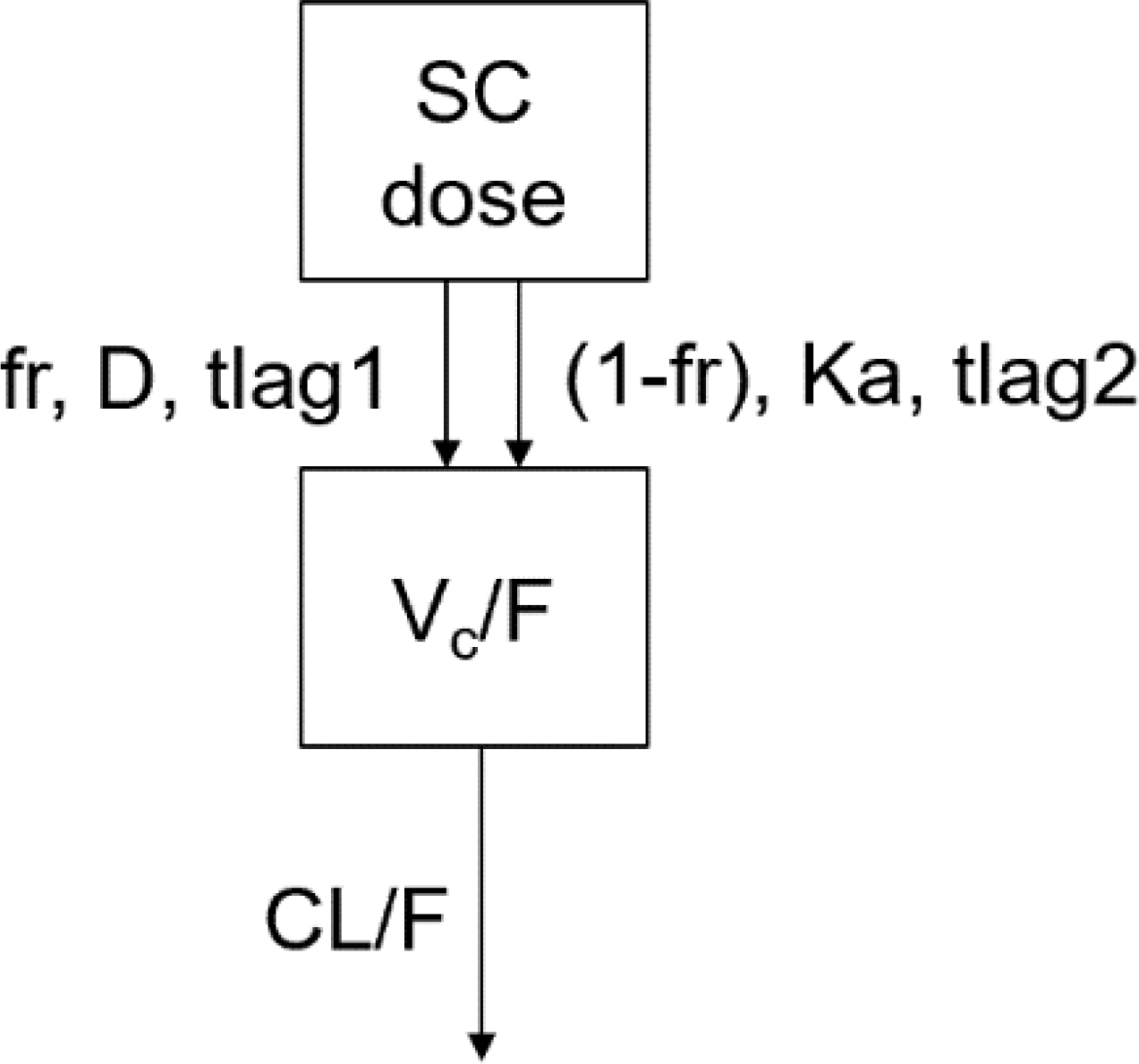


A one-compartment model with parallel zero-order and first-order absorption and first-order elimination from the central compartment was developed to describe the PK of PEG-rhGH in pediatric patients after SC administration.

### Patient Subjects for Clinical Trials

The Phase III clinical trial was a randomized, multi-center, open-label, parallel-controlled study in China. The subjects [short-stature children due to endogenous growth hormone deficiency (GHD)] were randomized 2:1 into each group, 240 in the Jintrolong^®^ treatment group (0.2 mg/kg/week; the PEG exposure is about 0.009 µmol/kg/week), and 120 in the control (daily rhGH Jintropin drug) group (0.25 mg/kg/week, no PEG). The trial was simultaneously performed at 6 centers, where 343 subjects were enrolled and had completed 6 months of treatment before statistical analysis.

The Phase IV clinical trial contained 4 sub-studies summarized as follows: (1) Sub-study 1 was a multicenter, randomized, parallel, dose-controlled clinical study: a low-dose Jintrolong^®^ group (0.12 mg/kg/week, corresponding to a PEG exposure of about 0.005 µmol/kg/week) and a high-dose Jintrolong^®^ dose group (0.20 mg/kg/week) where 600 subjects were randomized at a ratio of 1:1 into the aforementioned groups; (2) Sub-study 2 was a multicenter, randomized, parallel, dose-controlled clinical study with a low-dose Jintrolong^®^ dose group (0.14 mg/kg/week, a PEG exposure of about 0.006 µmol/kg/week) and a high-dose Jintrolong^®^ group (0.20 mg/kg/week) in which 950 subjects were randomized at a ratio of 1:1 into the above two groups; (3) Sub-study 3 was a multicenter, open-label clinical study in which the subjects received Jintrolong^®^ at the adjustable dose of 0.10–0.20 mg/kg/week with 900 total subjects; (4) Sub-study 4 was a multicenter, randomized, parallel clinical study of different dosing intervals, where a weekly Jintrolong^®^ dosing group (0.20 mg/kg/week), a biweekly Jintrolong^®^ dosing group (0.20 mg/kg/2week) and a short-acting rhGH Jintropin AQ control group (0.25 mg/kg/week, no PEG, once a day) were compared in 900 subjects randomized at a ratio of 1:1:1 into the above three groups. The overall treatment courses were 26 weeks in duration throughout all four sub-studies.

### Statistical Analysis

For preclinical studies in cynomolgus monkeys, all statistical tests were conducted as two-sided tests, and the level of significance was set at 0.05. Group means and standard deviations were calculated in the control group and test article-treated groups. Data for each sex within a set were analyzed separately. The data were analyzed with the following procedures: a Levene’s test was performed to test for variance homogeneity. A one-way analysis of variance (ANOVA) was performed when the variances were not significantly different (*p >*0.05). When ANOVA showed significance (*p ≤*0.05), a Dunnett’s test was performed for multiple comparisons. A Kruskal–Wallis test was performed in the case of heterogeneity of variance at *p ≤*0.05, a Kruskal–Wallis test was performed. When the Kruskal–Wallis test showed significance (*p ≤*0.05), a two-independent-samples test (Mann–Whitney U) was performed for multiple comparisons. When the number of samples was smaller than 3, the data were excluded from the statistical analysis. The independent-sample t-test was conducted when the data were analyzed only between Group 1: control group and Group 3: 1 mg/kg Jintrolong^®^ group.

For clinical studies of pediatric patients with GHD, summary statistics (n, mean, standard deviation, median, minimum, and maximum values for continuous variables, and number [%] of subjects in each category for categorical variables) were analyzed by the treatment group. All safety parameters (including AEs, vital signs, and laboratory evaluations) were summarized using the safety analysis (SA) set. No formal hypothesis testing was performed. All AEs were coded using MedDRA. All TEAEs were summarized and presented in the listings by the number of subjects reporting an event, the percentage of subjects with that event, the number of events, the grade, and the relationship to treatment. Percentages were calculated based on the number of subjects who received the treatment during the study.

## Results

### Vacuolation of CP was Observed Following 26-Week Administration of 1 mg/kg/week of Jintrolong^®^ and was not Reversible After 26-Week Recovery

Cynomolgus monkeys received subcutaneous injections of 1 mg/kg/week Jintrolong^®^ or excipient control once weekly for 26 consecutive weeks, followed by a 26-week recovery period to investigate the possible vacuolation of the CP and the potential for reversibility following recovery in non-human primates treated with Jintrolong^®^.

As shown in **Table 2**, at the 26-week terminal necropsy (Day 184), minimal to moderate vacuolation of epithelial cells of the CP was noted in 5/6 animals (2 males and 3 females) at 1 mg/kg/week at the interim necropsy, with minimal vacuolation in 4/6 animals and moderate vacuolation in 1/6 animals. Vacuolation in the CP was measured at the 26-week recovery necropsy (Day 366) after a 26-week PEG-rhGH administration to explore the reversibility of CP vacuolation. Minimal to moderate vacuolation of epithelial cells of the CP was still noted in 4/4 animals (2 males and 2 females) at 1 mg/kg/week, with minimal vacuolation observed in 3/4 animals and moderate vacuolation observed in 1/4 animals. The incidence and severity of CP vacuolation in these animals were similar compared to the degree of CP vacuolation identified at the 26-week recovery necropsy (Day 184), suggesting that vacuolation in CP was irreversible within 26-week recovery. These data revealed that repeated subcutaneous injection of Jintrolong^®^ at 1 mg/kg/week for 26 consecutive weeks resulted in minimal to moderate vacuolation of epithelial cells of CP in cynomolgus monkeys and was irreversible after a 26-week recovery. Corresponding anti-PEG IHC was also processed to investigate whether the vacuolation in CP resulted from PEG accumulation or PEG-related in CP cells. **Table 2** shows that all 6/6 animals with vacuolation in CP exhibited IHC-positive for PEG, indicating that the vacuolation was likely PEG-related. However, no PEG was detected in the CP of the 6 animals after a 26-week recovery.

**Table 2:** H&E staining and anti-PEG IHC staining counts of CP epithelium with 26-week administration and extra 26-week recovery.

Group	PEG-rhGH (mg/kg/week)	H&E staining 26-week terminal necropsy (Day 184),	H&E staining 26-week recovery necropsy (Day 366)	Anti-PEG IHC 26-week terminal necropsy (Day 184),	Anti-PEG IHC 26-week recovery necropsy (Day 366)
Data collection					
Excipient control	0	-: 6/6	- : 4/4	-: 6/6	- : 4/4
PEG-rhGH	1	- 1/6; + : 4/6; ++:1/6	+ : 3/4; ++: 1/4	+: 6/6	-: 4/4

-: no vacuolation;

+: minimal vacuolation;

++: moderate vacuolation.

**Figure 1:**
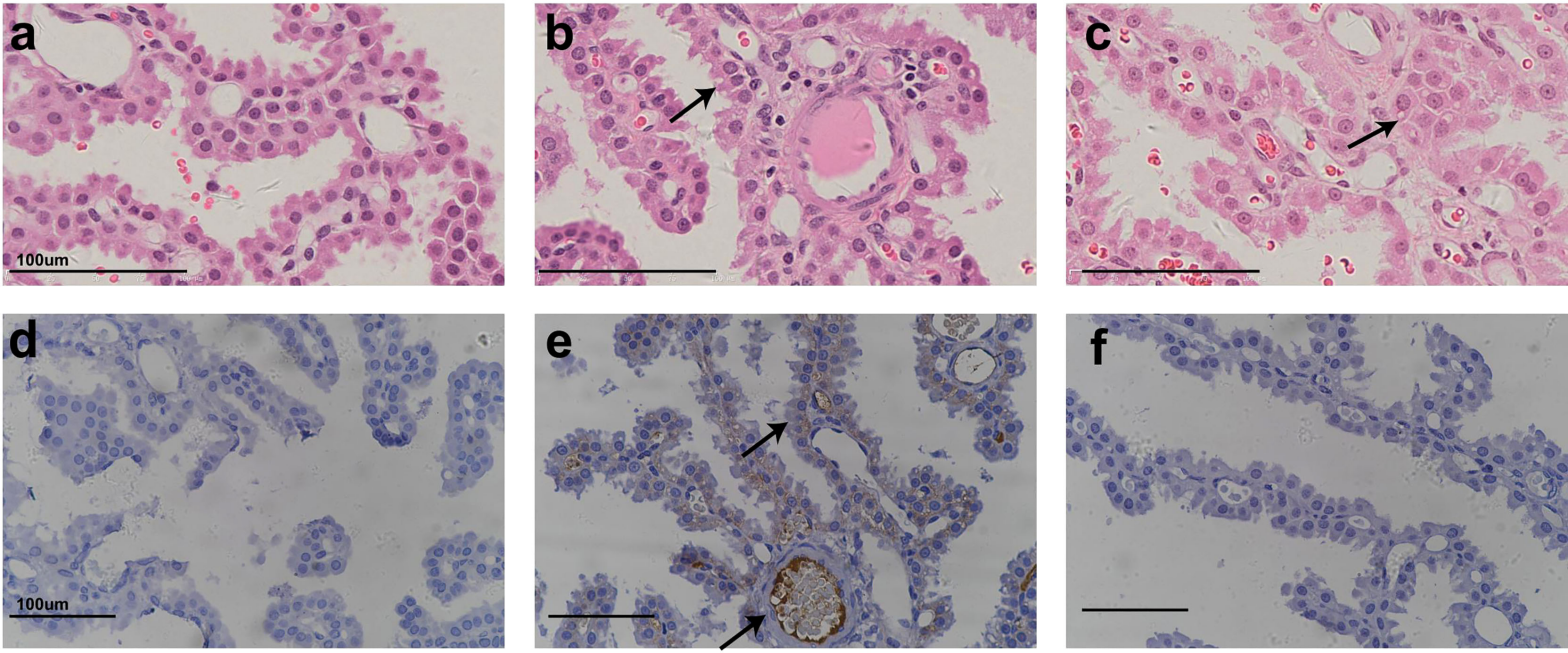
H&E staining and IHC staining of cynomolgus monkeys CP epithelium with 26-week administration of Jintrolong. **(A)** Excipient control group with 26-week administration. **(B)** Jintrolong group with 1 mg/kg/week administration for 26 weeks. **(C)** Jintrolong recovery group with 1 mg/kg/week administration for 26 weeks and following recovery period of 26 weeks. **(D)** anti-PEG IHC stain of control group. **(E)** anti-PEG IHC stain of Jintrolong group with 26 weeks of administration. **(F)** anti-PEG IHC stain of Jintrolong group after recovery. Arrows in **(B, C)** indicate cytoplasmic vacuoles in CP epithelium. Arrows in **(E)** indicate representative anti-PEG staining. Black line: under dose 3mg/kg/week; blue line: under dose 1mg/kg/week; red line: under dose 0. 3mg/kg/week; Dotted line: predicted threshold dose to induce vacuolation.

**Figure 1** shows representative histopathological examples of vacuolation (**Figures 1B, C**) and anti-PEG IHC staining images (**Figures 1D–F**) of CP epithelium with a 26-week administration of Jintrolong^®^ and following the 26-week recovery period. Positive anti-PEG was observed in the CP of the 26-week terminal necropsy (Day 184), but no anti-PEG immunoreactivity was observed at the 26-week recovery necropsy (Day 366). These findings suggest that it takes more than 26 weeks to reverse vacuolation in the CP, but there is a decrease in PEG immunoreactivity after the 26-week recovery period.

### Jintrolong^®^ Induced Vacuolation in CP was Dose- and Time-Dependent

To further explore whether the vacuolation in CP was Jintrolong^®^-associated with dose- and time-dependent, and since Jintrolong^®^ will be administered to pediatric patients for several years, longer-term toxicity studies (i.e., to assess the effects of long-term administration on CP vacuolation and reversibility) were conducted in which Jintrolong^®^ injections were subcutaneously administered at 0.3, 1, and 3 mg/kg/week to cynomolgus monkeys weekly for 52 weeks. The lowest dose used in this study, 0.3 mg/kg, approximates the maximum Jintrolong^®^ dose used in human clinical trials, while the 3 mg/kg dose is about ten-fold higher than the maximum dose used in humans.

As shown in **Table 3**, at the 52-week terminal necropsy (Day 374), minimal vacuolation of epithelial cells of the CP was observed in 2/8 animals of the excipient control group, while minimal vacuolation was observed in 1/8 animals at 0.3 mg/kg/week, indicating that the vacuolation in the 0.3 mg/kg/week group was within the control background. In the 1 mg/kg/week group, vacuolation was observed in 8/8 animals, with minimal vacuolation in 2/8 animals and moderate in 6/8 animals. In the 3 mg/kg/week group, vacuolation was observed in 8/8 animals, with moderate vacuolation in 5/8 animals and severe vacuolation in 3/8 animals. These findings suggest that the severity of Jintrolong^®^-induced vacuolation is dose-dependent.

**Table 3:** H&E staining counts of CP epithelium with 52-week administration at dose of 0.3, 1 and 3 mg/kg/week and after 104-/157- week recovery.

Group	PEG-rhGH (mg/kg/week)	PEG exposure per week (mg/kg)	52-week administration (Day 374)	104-week recovery (Day 1096)	157-week recovery (Day 1465)
Data collection					
Excipient control Group 1	0	0	-: 6/8; +: 2/8	-: 2/2	- :1/2; + :1/2
PEG-rhGH Group 2	0.3	0.55	-: 7/8; +:1/8	-: 2/2	-: 2/2
PEG-rhGH Group 4	1	1.82	+: 2/8; ++: 6/8	+: 2/2	+: 2/2
PEG-rhGH Group 5	3	5.45	++: 5/8; +++: 3/8	++: 2/2	++: 2/2

-: no vacuolation;

+: minimal vacuolation;

++: moderate vacuolation.

+++: severe vacuolation.

**Figure 2:**
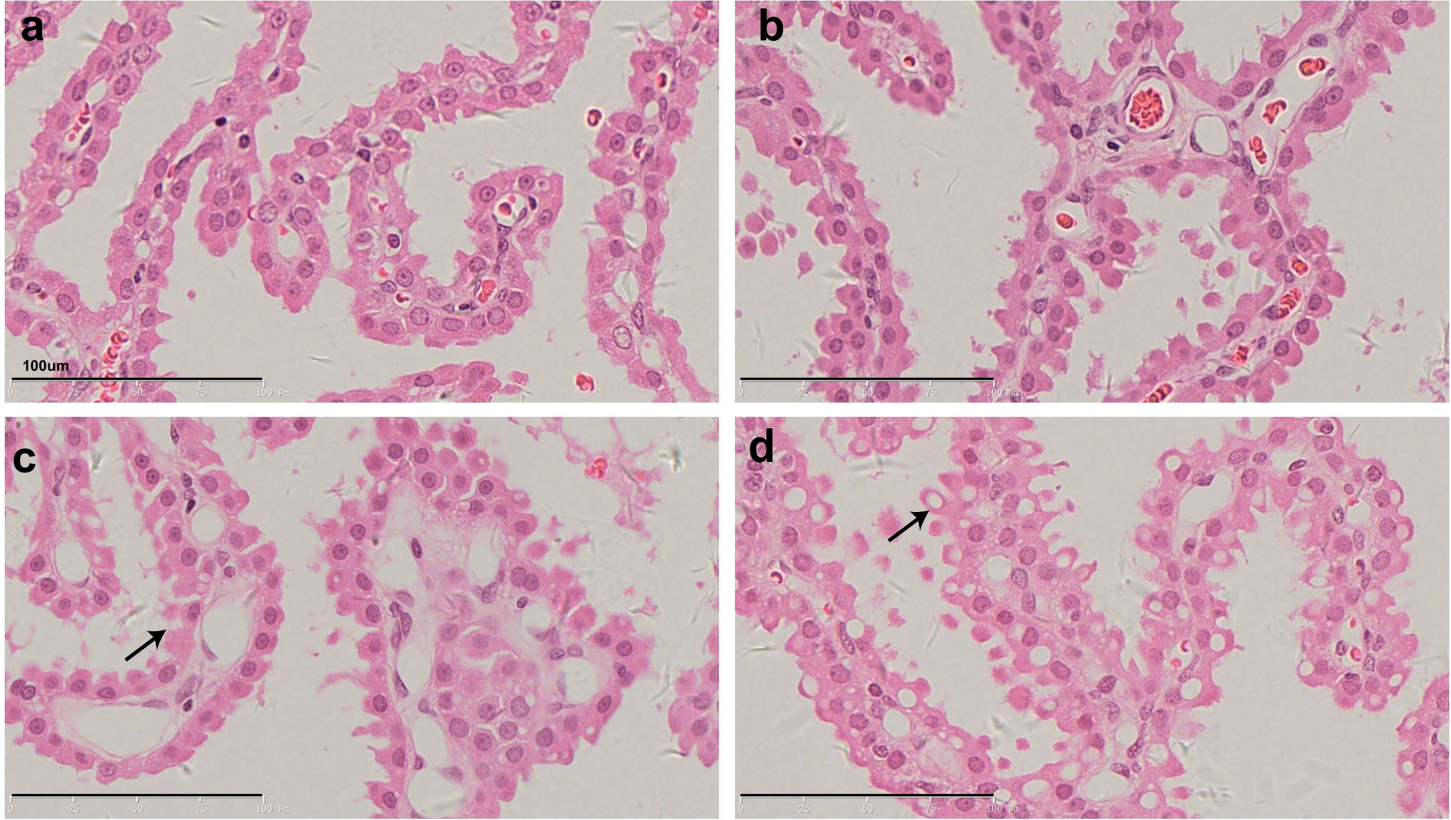
H&E staining of cynomolgus monkeys CP epithelium with 52-week administration of Jintrolong. **(A)** Excipient control group. **(B)** Jintrolong group with 0.3 mg/kg/week administration. **(C)** Jintrolong group with 1 mg/kg/week administration. **(D)** Jintrolong group with 3 mg/kg/week administration. Arrows in panels **(C)** and **(D)** indicate representative cytoplasmic vacuoles.

**Figure 2** shows representative histopathological images of H&E-stained CP epithelium following 52-week administration at doses of 0.3, 1, and 3 mg/kg/week, which also supports dose-dependent Jintrolong^®^-induced vacuolation.

In **Figure 2C**, animals that received 1 mg/kg/week of Jintrolong^®^ for 52 weeks exhibit severe CP vacuolation compared to the 26-week group at the same dose in **Figure 1B**, which indicates that Jintrolong^®^-induced vacuolation increases with time and underscores the need for long-term studies to examine the effects of Jintrolong^®^-induced CP vacuolation. Meanwhile, as shown in **Table 3**, 2/8 minimal vacuolation and 6/8 moderate vacuolation were observed at 1 mg/kg/week of Jintrolong^®^ for 52 weeks, which is more serious than the finding of 1/6 negative, 4/6 minimal vacuolation, and 1/6 moderate vacuolation under the same dose for 26 weeks in **Table 2**. It also suggests that the vacuolation is time-dependent.

All the histological slides were carefully checked. The vacuolation did not lead to any degeneration, necrosis, or inflammation in the CP cells or other brain tissue. Other tissues such as kidney, lung, heart, spleen, and liver tissue were processed, but no obvious vacuoles were detected throughout the study. Representative histopathological images under the control group and all three doses of the Jintrolong^®^ group are shown in **Figure S1**.

In addition, macrophages were checked in the lung, liver, spleen, and brain, but no vacuolation in macrophages was observed in all tissues throughout the study. Representative histopathological images under a high dose of 3 mg/kg/week of Jintrolong^®^ for 52 weeks are shown in **Figure S2**.

Since the vacuolation in CP was Jintrolong^®^ dose- and time-dependent, the vacuolation in CP was likely induced by Jintrolong^®^, which was further confirmed by the PEG presence in the vacuolation by EMT and IHC described afterward.

### Vacuolation in CP was Reversible in the 1 mg/kg Group After 104-Week and Partially Reversible in the 3 mg/kg Group After 157-Week Recovery

To evaluate the reversibility of PEG-induced vacuolation in the CP, the vacuolation after longer recovery periods (104 and 157 weeks) was evaluated following a 52-week administration of Jintrolong^®^.

As shown in **Table 3**, after the 104-week recovery period (Day 1,096), no obvious vacuolation of epithelial cells of CP in the brain was noted in animals (1 male and 1 female) treated with excipient control (group 1), but 1/2 animals treated with excipient control in the 157-week recovery displayed minimal vacuolation. In the low-dose 0.3 mg/kg/week Jintrolong^®^ group (group 2), no vacuolation was observed following the 104- or 157-week recovery periods. In animals treated with 1 mg/kg/week (group 4), minimal vacuolation of epithelial cells of CP in the brain was noted in 2/2 animals at both 104- and 157-week recovery time points. Since minimal vacuolation in the CP was still found (1/2) in the excipient control group at 157-week recovery and the severity of the vacuolation in 1 mg/kg/week (group 4) at the 104-week recovery decreased to the same background level as that in the excipient control group, the vacuolation in the 1 mg/kg/week at 104-week was completely reversible to the background of the control group after the 104-week recovery period. In animals treated with 3 mg/kg/week (group 5), moderate vacuolation (++) of epithelial cells of CP in the brain was noted in 2/2 animals. No severe vacuolation (+++) was observed following both the 104- and 157-week recovery periods, while 3/8 severe vacuolation (+++) was found at 3 mg/kg/week (group 5). These data indicate that vacuolation was only partially reversible in animals at 3 mg/kg/week after a 157-week recovery period.

**Figure 3:**
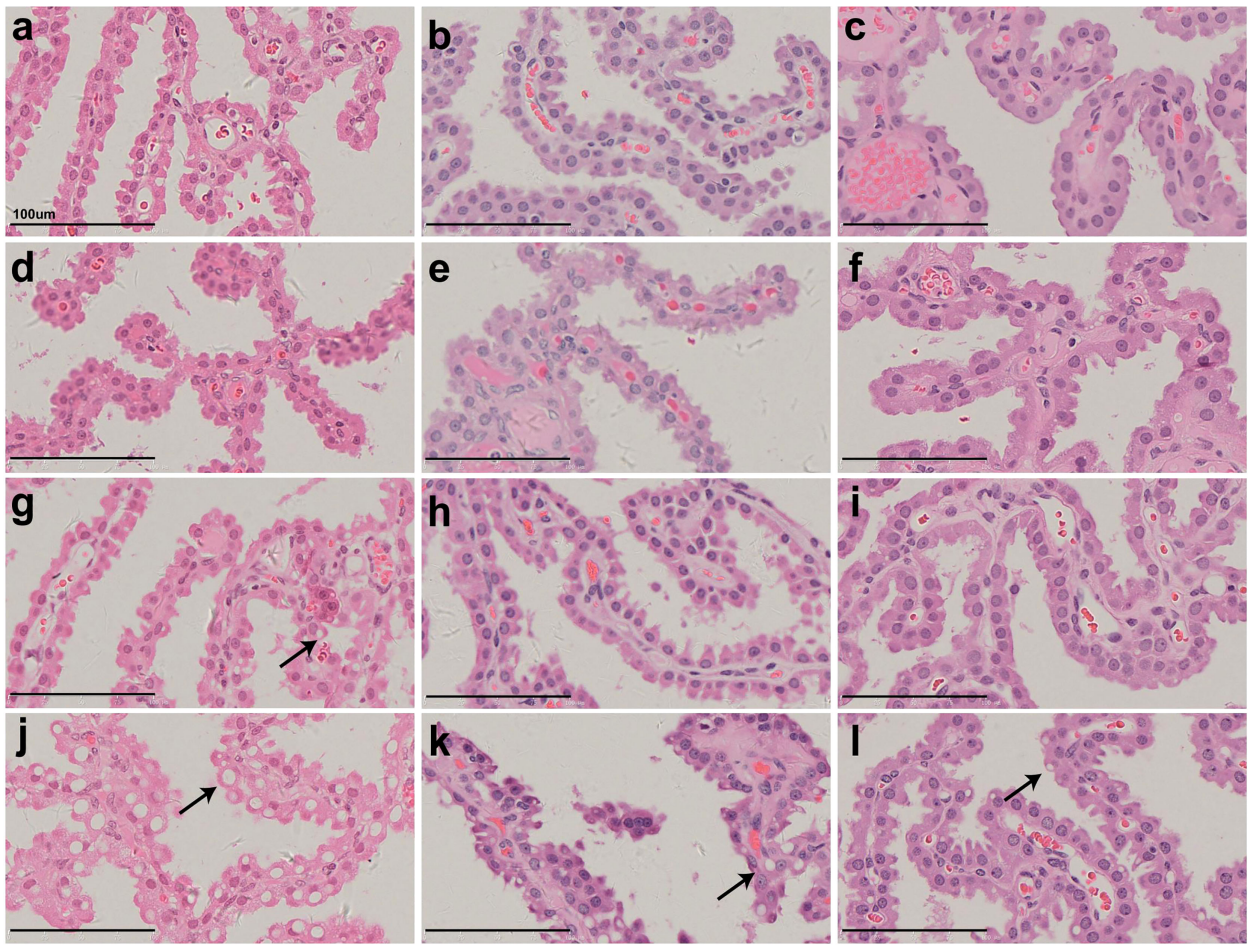
H&E staining of cynomolgus monkeys CP epithelium with 52-week administration of Jintrolong and after 104-/157- week recovery. **(A)** Excipient control group. **(B)** Excipient control group after 104-week recovery. **(C)** Excipient control group after 157-week recovery. **(D–F)** 0.3 mg/kg/week Jintrolong group at 52-week, after 104-week recovery, after 157-week recovery, respectively. **(G–I)** 1 mg/kg/week Jintrolong group at 52-week, after 104-week recovery, after 157-week recovery, respectively. **(J–L)** 3 mg/kg/week Jintrolong group at 52-week, after 104-week recovery, after 157-week recovery, respectively. Arrows indicate representative cytoplasmic vacuoles.

**Figure 3** shows representative pathological images of CP epithelium after 52- (**Figures 3A, D, G, J**), 104- (**Figures 3B, E, H, K**), and 157-week (**Figures 3C, F, I, L**) recovery period following administration of Jintrolong^®^ at dose of 0 (**Figures 3A–C**), 0.3 (**Figures 3D, E**), 1 (**Figures 3G–I**), and 3 (**Figures 3J–L**) mg/kg/week. Compared with 52-week terminal necropsy animals and 104-week recovery animals, the number and size of vacuoles decreased, indicating that vacuolation was at least partially reversible.

Further quantification of the vacuolation ratio/size in CP treated with different doses of Jintrolong^®^ at 52-week, recovery at 104-week, and 157-week was processed, further analyzed, and summarized in **Figure 8**.

### Vacuolation in CP correlated with PEG detection *via* IHC

**Figure 4:**
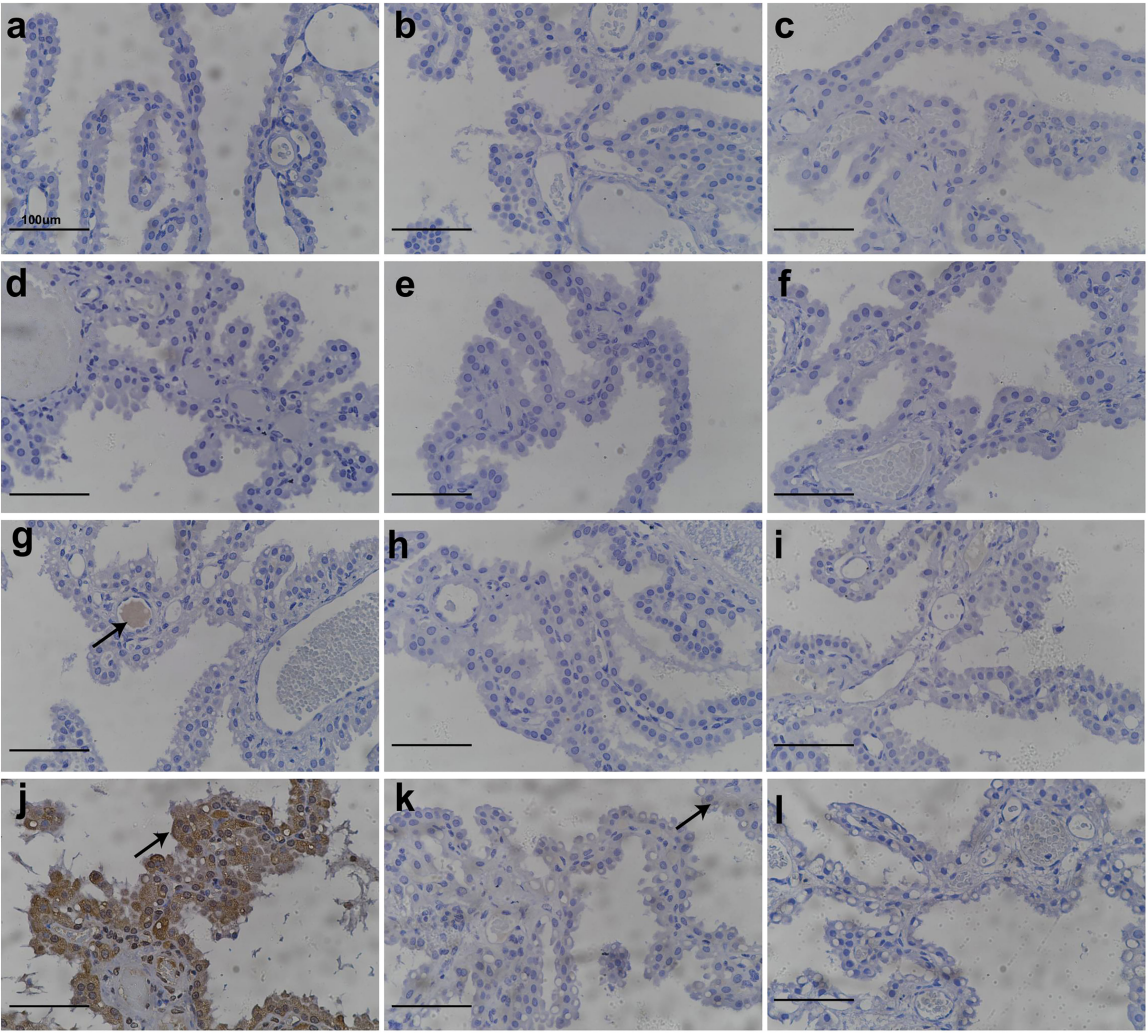
Anti-PEG IHC staining of cynomolgus monkeys CP epithelium with 52-week administration of Jintrolong and after 104-/157- week recovery. **(A)** Excipient control group. **(B)** Excipient control group after 104-week recovery. **(C)** Excipient control group after 157-week recovery. **(D–F)** 0.3 mg/kg/week Jintrolong group at 52-week, after 104-week recovery, after 157-week recovery, respectively. **(G–I)** 1 mg/kg/week Jintrolong group at 52-week, after 104-week recovery, after 157-week recovery, respectively. **(J–L)** 3 mg/kg/week Jintrolong group at 52-week, after 104-week recovery, after 157-week recovery, respectively. Arrow in panel **(G)** indicates anti-PEG staining in vessel. Arrow in panel **(J)** indicates representative anti-PEG staining in CP epithelium. Arrow in panel **(K)** indicate weak anti-PEG staining in recovery slides.

IHC experiments to detect PEG were conducted to further examine the relationship between the administration of Jintrolong^®^ and the development of vacuolation in the CP. As shown in **Figure 4**, no PEG staining was detected in the brain CP in animals of the control group (**Figures 4A–C**) or the 0.3 mg/kg group (**Figures 4D–F**). However, positive anti-PEG was observed in animals at 1 mg/kg (**Figure 4G**) and with an even stronger staining signal at 3 mg/kg (**Figure 4J**). A weak positive anti-PEG IHC result was observed in only one animal at 3 mg/kg at the 104-week recovery necropsy (Day 1,096) and 157-week recovery necropsy (Day 1,465). The incidence and severity of anti-PEG IHC data were also consistent with the results of microscopic observation, suggesting that the vacuolation of brain CP is related to the PEG.

Whole-brain slides were checked. Positive PEG staining was only detected in CP and blood vessels (**Figure 4G**) but not in other brain tissues, indicating that PEG or Jintrolong^®^ was unlikely to cross the blood-brain barrier composed of CP cells and enter other brain tissue. This result also supports the fact that the function of the CP epithelial cells in helping maintain fluid pressure in CSF and the blood-brain barrier may be intact in these animals.

### Vacuolation in CP Epithelial Cells did not Lead to the Obvious Alteration of Cellular Ultrastructure

To determine whether the Jintrolong^®^-dependent vacuolation observed in CP resulted in the alteration of cellular ultrastructure and function of the CP, brain tissue was collected for TEM at the terminal necropsy following the last dosing and at the recovery necropsy.

**Figure 5:**
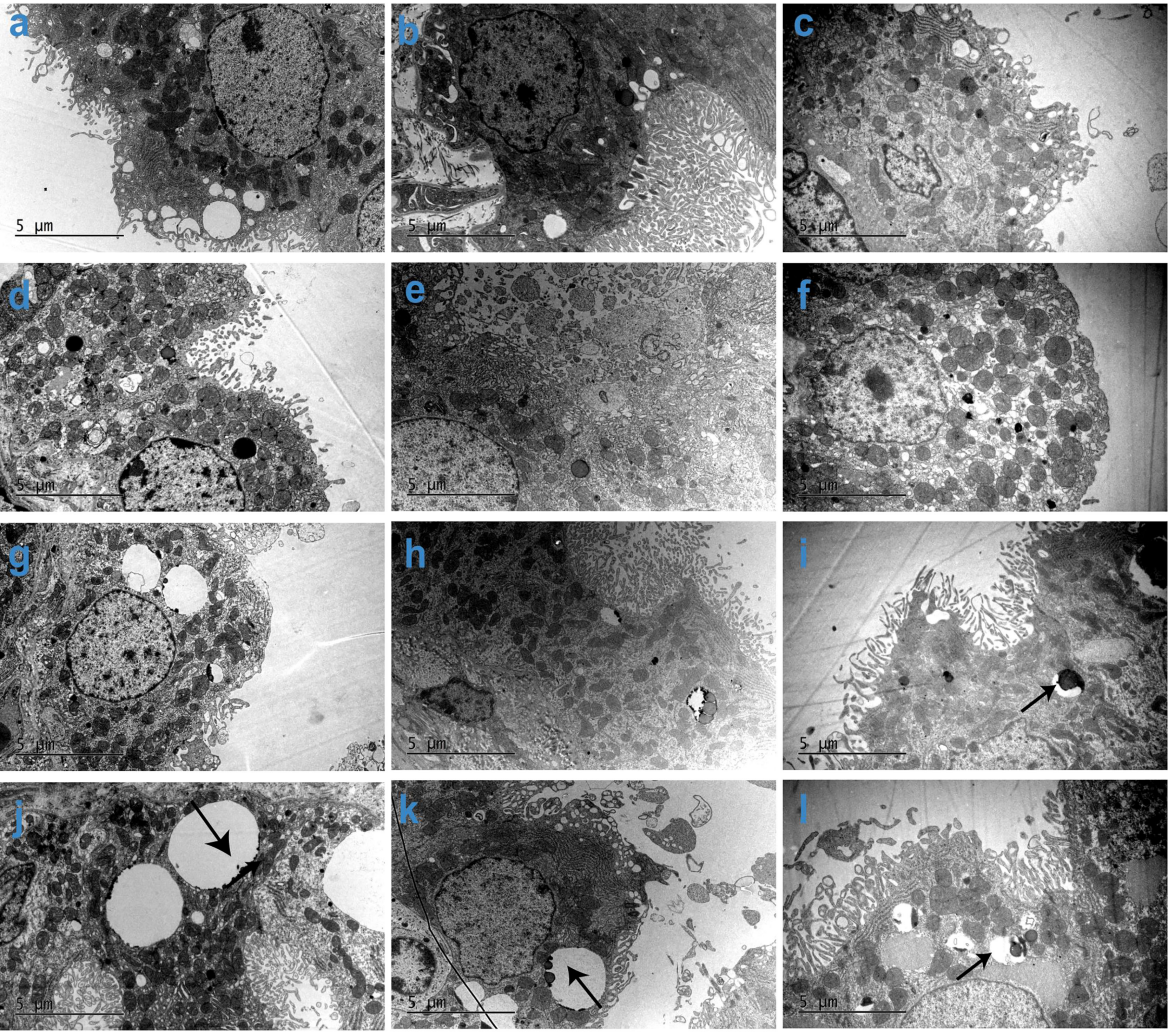
Subcellular imaging of cynomolgus monkeys CP epithelium with 52-week administration of Jintrolong and after 104-/157-week recovery. **(A)** Excipient control group with 52-week administration. **(B)** Excipient control group after 104-week recovery. **(C)** Excipient control group after 157-week recovery. **(D–F)** 0.3 mg/kg/week Jintrolong group at 52-week administration, after 104-week recovery, after 157-week recovery, respectively. **(G–I)** 1 mg/kg/week Jintrolong group at 52-week administration, after 104-week recovery, after 157-week recovery, respectively. **(J–L)** 3 mg/kg/week Jintrolong group at 52-week administration, after 104-week recovery, after 157-week recovery, respectively. Arrows in panels **(I–L)** indicate PEG in vacuoles.

As shown in **Figure 5**, the vacuolation observed by TEM is generally consistent with the pathological findings. As the dose increased, the vacuoles in CP epithelial cells were enlarged, more prevalent, and more likely to contain electron compacts, which manifest as dense black dots (denoted in **Figure 5** with arrows). These electron compacts are consistent with PEG deposits, as the location and shape of the compacts correlate with the pattern of anti-PEG IHC staining in the CP. Additionally, TEM examination of the brain CP also showed a trend toward recovery from vacuolation after a 104- and 157-week recovery period. In the same dose group, the 104-week recovery period group showed fewer and smaller vacuoles compared with the group without any recovery period, and the 157-week recovery group showed even fewer and smaller vacuoles.

The CP cells of each group under TEM were cubic, with abundant microvilli on the surface, and the tight junctions between the cells and the basement membrane on the bottom were intact. This result also supports the fact that the function of the CP epithelial cells in the blood-brain barriers is intact in these animals.

Animals in the low dose 0.3 mg/kg/week Jintrolong^®^ group did not show any cellular ultrastructure changes. Animals in the middle dose 1 mg/kg/week Jintrolong^®^ group only showed a slight ultrastructure change with fewer microvilli. Although some animals in the high dose 3 mg/kg/week Jintrolong^®^ group exhibited minor cellular ultrastructure changes such as fewer microvilli, minor mitochondrial swelling, and rough endoplasmic reticulum dilation, the nucleus and mitochondria had no obvious abnormalities, suggesting that the vacuolation did not lead to the distortion of the cytoplasmic or nuclear compartments. These minor changes were not sufficient to impact the cell viability and function.

Thirty-two animals were checked with TEM in this study. Minor mitochondrial swelling was observed only in 1 animal throughout the study. Rough endoplasmic reticulum dilation was only observed in 3 animals throughout the study, which were all from the high dose 3 mg/kg/week Jintrolong^®^ group (4 animals in this group). Representative cellular ultrastructure change figures are shown in **Figure S3**, compared to the control group (**Figure S3A**). The high dose 3 mg/kg/week Jintrolong^®^ group showed fewer microvilli, minor mitochondrial swelling, and rough endoplasmic reticulum dilation (**Figure S3C**), which was partially recovered in the 104-week (**Figure S3C**) and 157-week (**Figure S3D**) recovery periods.

Those alterations were quite slight/minor and recoverable, which indicates that they had no significant effect on cell viability and function.

### Vacuolation in CP Epithelium Did Not Lead to Significant Effect on Cell Proliferation

To determine whether vacuolation causes excessive cell proliferation, the expression of Ki-67 was detected by IHC. Ki-67 is a common proliferation marker used to identify non-G0 phase cells and can thus identify cells undergoing mitosis.

**Figure 6:**
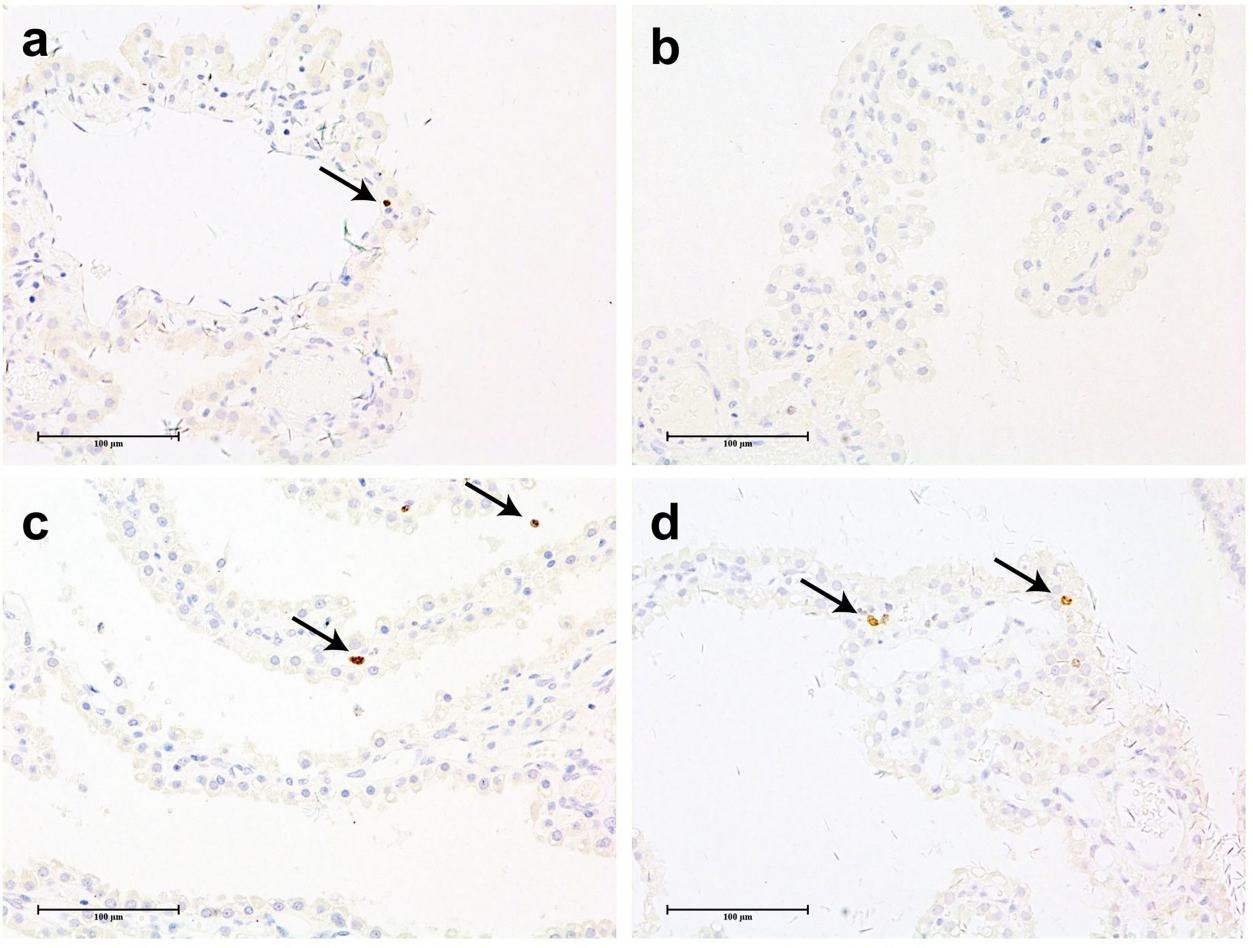
Anti-ki67 IHC staining of cynomolgus monkeys CP epithelium with 52-week administration of Jintrolong. **(A)** Anti-ki67 staining of excipient control group. **(B)** Anti-ki67 staining of 0.3 mg/kg/week Jintrolong group. **(C)** Anti-ki67 staining of 1 mg/kg/week Jintrolong group. **(D)** Anti-ki67 staining of 3 mg/kg/week Jintrolong group. Arrows in panels **(A, C, D)** showed representative cells with positive anti-Ki-67 staining. The scale bar is 100 µm.

**Figure 6** shows representative images of CP tissue stained with an antibody against Ki-67. There are only a few positive cells (+) or no positive cells (−) in the brain CP tissue of cynomolgus monkeys in most animals, except for one animal in the 3 mg/kg/week group, which had multiple positive cells in the CP(++). The number of positive cells in the brain CP tissue of cynomolgus monkeys in the drug administration group was relatively small and comparable with that of the excipient control group, which indicated that the administration of Jintrolong^®^ had no significant effect on the proliferation of cynomolgus monkey brain CP cells.

### Vacuolation in CP Showed no Significant Effect on CSF Composition and Glucose Metabolism in Brain of Cynomolgus Monkeys

Since vacuolation did not obviously affect the subcellular structure or cell proliferation of CP epithelial cells, the next question is whether the vacuolation in CP affected the function of CP epithelial cells and the brain. The indices of CP function measured were secreted CSF and cell metabolism of glucose.

CSF was collected and tested from monkeys treated with excipient control or Jintrolong^®^ (0.3, 1, or 3 mg/kg). There was no obvious dose-dependent trend noted in the concentrations of WBC, RBC, total protein (TP), albumin (Alb), and globulin (Glb) in the CSF of monkeys treated with Jintrolong^®^, although higher concentrations of these parameters were detected in individual animals at various individual time points. These outliers may be related to the contamination with blood during the CSF collection process and were not considered as test article related.

Jintrolong^®^ measurement by ELISA in CSF was also processed, but no Jintrolong^®^ was detected except for several samples with mixed blood during the CSF collection process, and the lower limit of quantification was 8 ng/ml, while the average C_max_ of Jintrolong^®^ in the serum concentration can reach 46,290 ng/ml in high dose group, which indicates that Jintrolong^®^ in the CSF was 6,157-fold lower than that in the serum. All these data suggest that Jintrolong^®^ in the CSF is quite limited.

**Figure 7:**
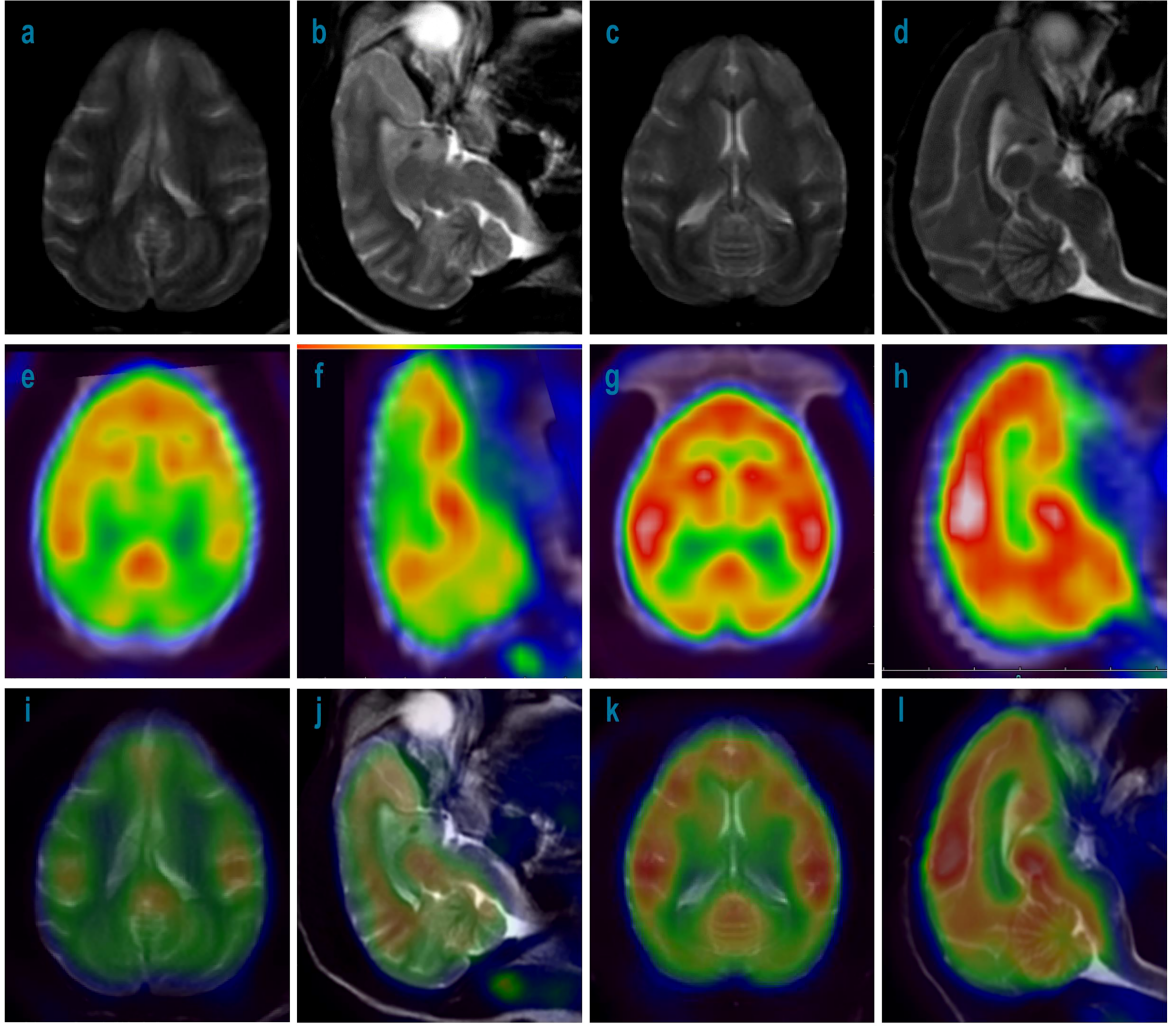
MRI and 18F-FDG-PET imaging of cynomolgus monkey brains with 52-week administration of Jintrolong and control after 148- week recovery. **(A)** MRI imaging for axial source of excipient control group. **(B)** MRI imaging for sagittal source of excipient control group. **(C)** MRI imaging for axial source of 3 mg/kg/week Jintrolong group. **(D)** MRI imaging for sagittal source of 3 mg/kg/week Jintrolong group. **(E–H)** corresponding 18F-FDG-PET imaging. **(I–L)** corresponding imaging merged by MRI and PET.

To determine if glucose metabolism in the brains of treated monkeys was intact, 18F-FDG-PET was performed in recovery animals treated with 3 mg/kg/week during the 148-week recovery period. As shown in **Figure 7**, compared with the control group, no detectable differences in glucose metabolism were found in the brain including CP in the 3 mg/kg treated group.

### Vacuolation in CP Showed no Significant Effect on Animal Neurobehavior

A functional observational battery (FOB) of neurobehavioral assessments was conducted to detect any neurobehavioral deficits resulting from potential CP dysfunction, including home cage observations, out-of-cage observations, and open field activity assessments in animals of both control and animals treated with 0.3, 1, or 3 mg/kg/week at the time point of last dosing (Day 368), the recovery period of 53 weeks (Day 739), and 104 weeks (Day 1095). The specific parameters are shown in **Table 4**. No abnormal findings were noted throughout the study, which indicated that vacuolation has no detectable effect on the neurobehavior of cynomolgus monkeys.

**Table 4:** Functional observational battery for neurobehavioral assessments.

Home cage observations	Test article-related changes	Out-of-cage observations	Test article-related changes	Open field activity assessments)	Test article-related changes
Data collection					
arousal	N	chaddock	N	Movement and Gait	N
posture	N	babinski	N	Proprioception (position sense)	N
tremors	N	Movement of the facial muscles	N	paresis	N
convulsions	N	Lacrimation	N	ataxia	N
fasciculations	N	sialorrhea	N	Slope assessment	N
Stereotypic behavior	N	Position of the eye lids	N		
Response to food	N	Pupillary light response Dysmetria Visual field Auditory response	N N N N		

Additionally, no mortality, moribundity, changes in clinical observations, or injection site changes were noted in animals treated with 0.3, 1, and 3 mg/kg/week throughout the study. No Jintrolong^®^-related changes were noted in body temperature, electrocardiogram parameters, blood pressure, ophthalmoscopic examinations, hematology, coagulation, clinical chemistry, urinalysis, or lymphocyte subpopulation in animals throughout the study. The parameters are listed in **Table S1**. The findings suggest CP vacuolation has no significant effect on the general health status of cynomolgus monkeys.

### Model-Based Prediction of Vacuolation Recovery in the CP of Cynomolgus Monkeys

While the vacuolation in CP was found to be completely reversible to normal in cynomolgus monkeys at 1 mg/kg/week after a recovery time of 104 and 157 weeks, only partial reversal of vacuolation in CP was observed at a high dose of 3 mg/kg/week. To predict how long it might take for CP vacuolation at 3 mg/kg to achieve complete recovery, the relative number and size of vacuoles in the pathology slides were quantitatively analyzed by the Halo AI tissue typing system. The quantitative evaluation data are roughly consistent with the semi-quantitative evaluation of pathologists.

**Figure 8:**
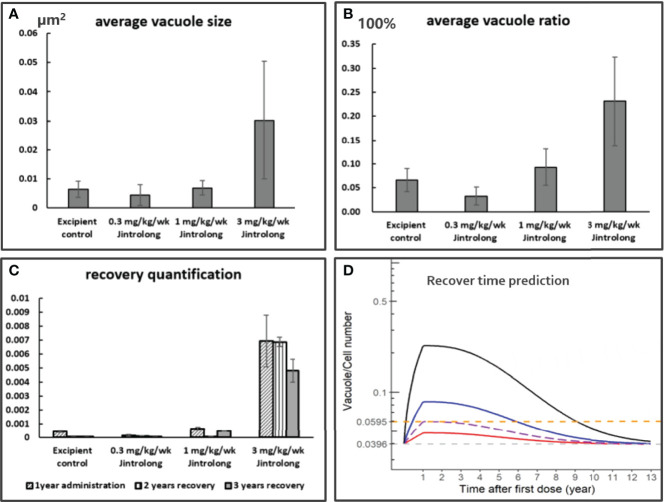
Quantification and Model based prediction of vacuolation recovery in the CP of cynomolgus monkeys. **(A)** The average vacuole size is dose-dependent, the y axis is the average vacuole size by µm^2^. **(B)** The average vacuole ratio is dose-dependent, the y axis is the average vacuole ratio in total CP epithelium. **(C)** The vacuolation recovery quantification by average vacuole area multiple average vacuole ratio of cells in CP epithelium. The vacuolation in CP epithelium is completely reversible in middle dose 1 mg/kg/week Jintrolong group and partially reversible in high dose 3 mg/kg/week Jintrolong group. **(D)** The modeling and simulation data are based on a pharmacokinetic modeling software package NONMEM (the pharmacokinetic modeling method shown in *Model Based Prediction of Vacuolation Formation and Recovery in the Cynomolgus Monkeys and in Clinical Pediatric GHD*) and the final graphical data analyzed with R programming. It was predicted to be 8.1 years for vacuolation in high dose 3 mg/kg/week Jintrolong^®^ group to get completely recovered in cynomolgus monkeys. The predicted minimum dose to induce vacuolation in monkey is 0.55 mg/kg/week. Black line: under dose 3mg/kg/week; blue line: under dose 1mg/kg/week; red line: under dose 0. 3mg/kg/week; Dotted line: predicted threshold dose to induce vacuolation.

As shown in **Figures 8A, B**, the severity of vacuolation in the CP epithelium increases with dose by both average vacuole size and average vacuole ratio (cells with vacuole/total CP epithelial cells). As shown in **Figure 8C**, no vacuolation was considered to be induced by Jintrolong^®^ in the low dose of 0.3 mg/kg/week Jintrolong^®^ group, as vacuolation was within the background vacuolation range in the excipient control group. Vacuolation was detected in the middle dose 1 mg/kg/week Jintrolong^®^ group but could be completely reversible with 104 weeks of recovery. Vacuolation in the CP epithelium was partially reversible in the high dose 3 mg/kg/week Jintrolong^®^ group, which was found to fit the relation with time and led to rough predictive vacuolation recovery data.

As shown in **Figure 8D**, a modeling and simulation strategy based on NOMEM was applied. PK and PD profiles for Jintrolong^®^ were simulated after subcutaneous injection in monkeys. Based on the model, it was predicted to take 8.1 years for vacuolation in the high dose 3 mg/kg/week Jintrolong^®^ group to get completely recovered in cynomolgus monkeys. The predicted minimum dose to induce vacuolation in monkeys is 0.55 mg/kg/week, which corresponds to 1.29 mg/kg/week in GHD children and is 6.45-fold higher than the clinical dose of 0.2 mg/kg/week Jintrolong^®^. The Vacuole/Cell Number in monkeys with 0.55 mg/kg/week Jintrolong^®^ administration in monkeys reached the median Vacuole/Cell Number (0.0595) observed at the end of 1-year treatment in the excipient control group. The mean AUC in the steady state of PEG-GH in a typical pediatric patient (32 kg) after 0.2, 0.4, and 0.6 mg/kg/week was comparable to that in monkeys after 0.08, 0.13, and 0.18 mg/kg/week, respectively. Based on this model, the clinical dose of 0.2 mg/kg/week of Jintrolong^®^ or even a 3-fold high dose of 0.6 mg/kg/week in PGHD patients will not reach the vacuole/cell number of 0.0595, suggesting that the possibility for vacuolation is quite limited.

### Safety Margin of Jintrolong^®^

Animals in the excipient group developed minimal vacuoles in the CP epithelium to a similar extent as animals in the low dose 0.3 mg/kg/week Jintrolong^®^ group, with a similar number and size of quantified vacuoles. Thus, CP vacuolation observed in animals of the excipient control group is considered as spontaneous vacuolation. The lack of detectable PEG IHC staining in the CP of the 0.3 mg/kg/week Jintrolong^®^ group despite observed vacuolation also suggests that no PEG accumulation in CP cells is found to cause vacuolation. Under the conditions of this study, the no observed effect level (NOEL) was determined to be 0.3 mg/kg. The most clinically relevant no observed adverse effect level (NOAEL) is determined to be 3 mg/kg as the vacuolation in CP cells did not elicit structural changes and is not expected to have resulted in functional impairment in cynomolgus monkeys.

In order to predict the safety in patients treated with Jintrolong^®^, we compared the monkey AUC and C_max_ at the NOAEL dose (3 mg/kg/week) with the human AUC and C_max_ at the clinical dose (0.2 mg/kg/week) in pediatric GHD patients to generate a safety margin.

In the 52-week repeated administration toxicity study in cynomolgus monkeys described herein, the serum concentration of Jintrolong^®^ was analyzed and the steady-state AUC_last_ in animals administered 3 mg/kg/week was 4,487,000 ± 850,370 ng ∗ h/ml and the C_max_ was 46,290 ± 7,570 ng/ml.

As shown in **Table 5**, compared to the AUC and C_max_ of a clinical dose of 0.2 mg/kg in the PGHD patients, the NOEL defined at 0.3 mg/kg Jintrolong^®^ of TK exposures in the non-human primate (NHP) study exhibited safety margins greater than 4.9 and 9.3 based on the AUC and C_max_, respectively, indicating that no vacuolation or PEG accumulation will occur in the pediatric GHD patients at the clinical dose of 0.2 mg/kg/week. While the TK exposure for monkeys at 3 mg/kg Jintrolong^®^ was defined as NOAEL, it exhibited safety margins greater than 120.5 and 136.1 based on the AUC and C_max_, respectively, indicating a huge safety margin of the clinical dose in PGHD patients based on the nonclinical NOAEL.

**Table 5:** Safety Margins for the proposed clinical doses.

	C_max_ (ng/mL) ±SD	AUC_last_ (ng*hr/ml) ±SD	MoE C_max_	MoE AUC
Data collection				
In cynomolgus monkeys 0.3 mg/kg QW	3170 ± 1380	182550 ± 62770	**9.3**	**4.9**
In cynomolgus monkeys 1 mg/kg QW	13480 ± 2710	1120960 ± 196060	39.6	30.1
In cynomolgus monkeys 3mg/kg QW	46290 ± 7570	4487000 ± 850370	**136.1**	**120.5**
In children with GHD 0.2 mg.kg QW	340 ± 44	37229 ± 7911	NA	NA

A GLP compliant 52-week repeat-dose toxicity study in monkeys MOE (Multiple of Exposure) was calculated as ratio of serum PK parameters in the pivotal toxicity study and the clinical dose 0.2 mg hGH/kg/week of Jintrolong in GHD children.

### Adverse Reactions Reported in Clinical Trials of Jintrolong^®^

To further assess the safety of Jintrolong^®^ in pediatric GHD, adverse reactions reported in Phase III and Phase IV clinical trials were analyzed. The Phase III clinical trial was a randomized, multi-center, open-label, parallel-controlled trial with 360 subjects. They were randomized into two groups: the Jintrolong^®^ treatment group (0.2 mg/kg/week, 240 subjects) and the Jintropin (short-acting rhGH) control group (0.25 mg/kg/week, 120 subjects). The incidence of AR was 31.58% in the Jintrolong^®^ treatment group and 22.61% in the control group over the span of 25 weeks, with no statistically significant difference in the incidence of AR between the two groups (p >0.05). The ARs included peripheral edema, thyroid hypofunction, arthralgia, and discomfort at the injection site, which are common adverse reactions to the use of rhGH and similar to those reported in the Phase II clinical trial (21). Most of the adverse events that occurred in the trial were mild and required no treatment discontinuation. Considering that in cynomolgus monkeys, PEG-induced vacuoles are formed in CP following six weeks of Jintrolong^®^, the PEG-related ARs should be related to the central nervous system (CNS) and theoretically increase over time. No new ARs, including those that would be associated with CP vacuolation, were observed in the Phase III clinical trial. Headache, dizziness, acute otitis media, drowsiness, attention deficit, and febrile seizures were considered to be CNS-related ARs in this study. However, very rare incidences of CNS-related ARs were reported, and the incidence of ARs and CNS-related ARs in each group gradually decreased with the extension of the treatment time. As shown in **Table 6**, the incidence of ARs of the Jintrolong^®^ group decreased from 25.88 to 10%, while the incidence of CNS-related ARs of the Jintrolong^®^ group decreased from 0.88 to 0% over the span of 25 weeks.

**Table 6:** Adverse reactions(ARs) reports analysis in clinical study.

Phase III	Patients No	AR No	CNS related AR No	AR%	CNS-AR%
Data collection					
Jintrolong 0.2 mg/kg QW	228	72	4	31.58	**1.75**
Jintropin 0.25 mg/kg QD	115	26	1	22.61	0.87
Jintrolong 1-4 week	228	59	2	25.88	0.88
Jintropin 1-4-week	115	15	0	13.04	0
Jintrolong 5-13 -week	226	41	2	18.14	0.88
Jintropin 5-13 -week	115	14	1	12.17	0.87
Jintrolong 14-25-week	220	22	0	10	0
Jintropin 14-25-week	114	10	1	8.77	0.88
**Phase IV-I**	**Patients No**	**AR No**	**CNS related AR No**	**AR%**	**CNS-AR%**
Data collection					
Jintrolong 0.2 mg/kg QW	686	74	5	**10.79**	**0.73**
Jintropin 0.25 mg/kg QD	91	9	0	9.89	0
**Phase IV-2**					
Jintrolong <0.15 mg/kg QW	82	3	0	3.66	0
Jintrolong 0.15-0.17 mg/kg QW	186	13	1	6.99	0.54
Jintrolong 0.17-0.2 mg/kg QW	153	22	1	14.38	0.65
Jintrolong 0.2 mg/kg QW	269	22	2	8.18	0.74
Phase IV Follow-up*– Jintrolong 0.2mg/kg QW					
	**Patients No**	**AR No**	**CNS related AR No**	**AR%**	**CNS-AR%**
Phase IV-1^st^ year	644	64	3	9.94	0.47
Phase IV-2^nd^ year	411	9	1	2.19	0.24
Phase IV-3^rd^ year	401	5	0	1.25	0

* Only adverse events that occurred within the year were analyzed, and the denominator is the number of subjects still under observation in that year.

In the Phase IV clinical trial, the efficacy and safety of Jintrolong^®^ in 3,000 pediatric GHD patients were investigated with four sub-studies. The incidence of ARs was 10.79% in the Jintrolong^®^ group (Group A) and 9.89% in the Jintropin control group (Group E). There was no statistically significant difference between these 2 groups (p >0.05), which suggests that these ARs are not related to the administration of PEG. No new adverse reactions were observed in the phase IV clinical trial, and most of the adverse events in the trial were mild. Meanwhile, CNS-related ARs were very rare. As shown in **Table 6**, CNS-related ARs were reported in 5 patients of the Jintrolong^®^ group (Group A) with an incidence of 0.73%, while none were reported in the Jintropin control group (Group E). Although the incidence of ARs and CNS-related ARs increased with increasing dose, only one or two events in each group were reported, with an incidence of less than 0.74%. Additionally, all reported CNS-related ARs were considered mild and recoverable, which included 3 incidences of dizziness, 1 headache, and 1 tremor.

In the phase IV follow-up study with a treatment time of 3 years in 644 patients, 4 CNS-related ARs were reported, including 1 headache, 2 dizziness, and 1 drowsiness. As shown in **Table 6**, three of these ARs were reported in the first year. The incidence of ARs and CNS-related ARs gradually decreased with the extension of the treatment time. All these data indicate that the CNS-related ARs are not related to PEG accumulation in CP since the CNS-related Ars did not gradually increase over time but, in contrast, gradually decreased. Furthermore, we previously predicted in the safety margin section that clinically no vacuolation and PEG accumulation would occur at a clinical dose of 0.2 mg/kg/week.

All these clinical safety data are consistent with the published phase II 24-month follow-up study data and support that Jintrolong^®^ can increase convenience and compliance in GHD children without increasing safety concerns.

## Discussion

Most pegylated pharmaceuticals induce vacuolation in animal tissues and macrophages in nonclinical studies due to PEG accumulation. Currently, although the accumulation of PEG in the choroid plexus epithelium of animals administered repeat-doses of PEGylated products appears to be an adaptive, non-adverse finding (41) based on the lack of any signs of tissue degeneration or dysfunction, safety concerns about the PEG accumulation (28) and PEG-induced vacuolation persisted due to the lack of extensive investigation and data on the impact on tissue function, particularly in the CP tissue function and epithelial cellular structure by EMT, etc. Most importantly, the safety concerns could not be truly addressed due to the lack of clinical safety data generated from large patient populations with long-term PEGylated product treatment. In this study, we provided extensive nonclinical data on the CP vacuolation and large clinical safety data to support the long-term use of the Jintrolong^®^ in pediatric GHD patients.

In our nonclinical study, vacuolation was detected only in epithelial cells of the CP in cynomolgus monkeys but not in brain macrophages because macrophages are rarely observed in the brain of normal cynomolgus monkeys. Reports in the literature have shown that the CP only supports the entry of monocyte-derived macrophages (MDMs) into the CNS under specific circumstances, such as in animal models of traumatic brain injury, spinal cord injury, and Alzheimer’s disease (42). The reason why vacuolation in the kidney or macrophages was not detected in cynomolgus monkeys of our study could be that the doses used in our study were not high enough. As shown in the study by Fletcher et al., non-adverse levels of vacuolation were observed in monkey macrophages and epithelial cells in the CP and kidney with doses of 24 mg/kg/week for 3 months (33).

Notably, the vacuolation of epithelial cells of brain CP was found to be reversible or partially reversible in a dose-dependent manner in our nonclinical study. This result is consistent with the review by Ivens et al. and further supports that the formation of cellular vacuoles is reversible given sufficient recovery time after the vacuolation-inducing agent was removed (15, 43). The main contributor that determines the rate of PEG accumulation in cells and subsequently induces vacuolation is the slow metabolism of PEG and the turnover rate of the cells. The ether linkages between ethylene glycol subunits in PEG molecular are biologically stable because of the lack of mammalian etherases (44). Jintrolong^®^ contains methoxy-capped PEG, which renders the oxidation of terminal hydroxyl to carboxyl groups of PEGs caused by alcohol dehydrogenase negligible (45). PEG chain degradation has been observed to be slow (46, 47), and degraded PEG is eliminated slowly through the kidney or excreted through the bile to avoid high molecular weight PEG accumulation when PEG is administered in small amounts (48). There are also publications proposing the hypothesis of “reputation,” which claims that rod-shaped and stiff molecules can pass through renal glomeruli even at higher molecular sizes (45). The clinical data about the PEG moiety of Certolizumab Pegol show that the free PEG molecule is not further metabolized to lower molecular weight forms but is rapidly excreted through the kidneys (49). The reversibility of PEG-induced vacuolation thus depends on the rate of clearance of PEG from the cells. As mentioned in Baumann’s publication, the mechanism of how PEG is released from cells into the bloodstream has not been confirmed, but may occur through exocytosis or cell turnover (45). PEG and PEG metabolite detection in urine and plasma is particularly important in further nonclinical and clinical studies to quantify PEG metabolism and excretion. The analysis of PEG in whole animals or humans is technically difficult because the amount of PEG in urine is quite low and PEG metabolites are diverse mixtures with a wide range of varying chain lengths. The liquid chromatography-tandem mass spectrometry (LC-MS/MS) was used to analyze a 60 kDa PEGylated drug (BAY 1025662) in the urine of male Wistar rats treated with 11 mg/kg of the PEG molecule (47). The calibration ranged from 0.1 to 10 mg/L in urine after a single intravenous administration of the PEGylated drug. LC-MS/MS was also applied to analyze the 40 kDa PEGylated drug N8-GP in rats and humans (50). Further exploration of Jintrolong^®^ is still needed to detect intact PEG and lower doses or metabolites of PEG.

No PEG IHC staining was detected in the brain parenchyma, suggesting that there was no potential impact on brain parenchyma function. No positive PEG-rhGH was detected in CSF samples, indicating that the chance for PEG or Jintrolong^®^ to cross the blood-brain barrier is quite limited. These data are consistent with the reported results of the distribution of a 60 kDa PEG moiety in BAY 94-9027, in which radiolabeled PEG was detected in very low amounts in the brain (47, 51, 52). Importantly, the PEG-related vacuolation did not lead to increased CP cell proliferation, apoptosis, or glucose metabolism, suggesting that vacuolation did not affect the essential functions of CP cells.

Despite the intensive research on cytotoxic induction of vacuolation and its downstream effects, there is no definitive mechanism or functional impact of vacuolation (15, 16). Theoretically, significant cellular vacuolation could result in the disruption of the cytoarchitecture, leading to compromised function of tissues, such as epithelial cells in the CP. The main function of CP epithelial cells is to secrete CSF (53), which is an important physiological substance that maintains the homeostasis of the CNS. In our studies, no functional or cellular consequences of vacuolation were observed in the CP of epithelial cells at any dose of PEG, suggesting that the PEG-induced cellular vacuolation was benign.

Animals in the 0.3 mg/kg/week Jintrolong^®^ group developed vacuoles to a similar extent compared to animals in the excipient control group. Thus, under the conditions of this study, the NOEL was determined to be 0.3 mg/kg/week. The NOAEL was determined to be 3 mg/kg/week as the vacuolation in CP cells did not elicit structural changes and is not expected to have resulted in functional impairment in cynomolgus monkeys. The TK exposure for monkeys at 3 mg/kg exhibited safety margins greater than 120.5 and 136.1 based on the AUC and C_max_, respectively, indicating a good safety margin of the clinical dose in the PGHD patients with minimal vacuolation concern.

The neurobehavioral parameters in cynomolgus monkeys were monitored to assess the CNS-related functional change induced by vacuolation in CP, but no differences were observed between animals treated with control or Jintrolong^®^. In clinical trials, Phase III (360 patients) and Phase IV (3,000 patients) studies with 26 weeks of treatment demonstrated no statistically significant difference in the incidence of ARs between the Jintrolong^®^ group and the daily rhGH injection control group. A follow-up study of the Phase IV with treatment time as long as 3 years in 644 patients was also investigated, and no obvious differences in the incidence of ARs and CNS-related ARs between the treatment groups were found. These data are consistent with other PEG-conjugated drug clinical trials, such as those conducted for 40 kDa PEG N9-GP (50, 54, 55) and 60 kDa PEG BAY 94-9027 (56). In a long-term safety study of N9-GP administered for at least 5 years in 25 children with hemophilia B from 17 sites in 8 countries, the mean PEG plasma reached a steady state after 6 months, and no new abnormal findings in neurological examinations were observed in the study (55).

A recent publication by Carcao et al. shows some neurological examination data in the safety and efficacy studies for N9-GP treated children with hemophilia B for at least 5 years, including general appearance, language skills, social skills, and head circumference; no abnormal findings in neurological parameters were detected (55). The results are consistent with our clinical safety data and support PEGylated pharmaceuticals such as Jintrolong^®^ for long-term use at a clinical dose. To the best of our knowledge, this is the first study to report the extent and reversibility of CP vacuolation caused by the administration of a PEGylated rhGH drug in monkeys, with an administration time as long as 52 weeks and a recovery time as long as 157 weeks (10, 16). Our data showed that repeated subcutaneous injections of Jintrolong^®^ at 1 mg/kg/week for 26 consecutive weeks resulted in minimal to slight vacuolation of epithelial cells of CP in cynomolgus monkeys and that Jintrolong^®^-induced vacuolation in the CP is dose- and time-dependent, which is consistent with other reports from different research groups (5, 10, 16).

In addition, we report clinical safety assessment results spanning 3 years in more than 644 GHD pediatric patients. The PEG weekly dose level administered to GHD children at the clinical dose was 10-fold below the theoretical threshold (3.7 mg PEG (40 kDa)/kg/QW or 0.4 µmol PEG/kg/month) recommended by the Committee for Medicinal Products for Human Use (CHMP 2012) for CP epithelial cell vacuolation. Based on the NOAEL and AUC, the clinical dose of 0.2 mg/kg/week of Jintrolong^®^ (corresponding to 0.009 µmol/kg/week of PEG) exhibits safety margins greater than 120.5-fold, which is predicted to be a minimum-to-no risk for inducing vacuolation in GHD pediatric patients. We are continuing to collect adverse effects data for longer administration times, as patients with congenital panhypopituitarism or other forms of GHD may need lifelong growth hormone replacement therapy. We also plan to conduct the neuropsychological battery study in clinical settings in the near future.

Based on Lonapegsomatropin (ACP-011, Skytrofa^®^) non-clinical document (57), 90% of PEG will be cleared from the systemic circulation in GHD children within approximately 3 months from the end of treatment (based on the predicted systemic half-life of 26 days) and 90% of PEG will be cleared from the choroid plexus within 16 months (based on the predicted half-life of approximately 5 months). These data support the notion that PEG could be cleared from the body at a certain rate. Considering that Skytrofa^®^ is already approved by the FDA and represents minimal-to-no risk to GHD children under its clinical dose of 0.24 mg/kg/week (corresponding to 0.011 µmol/kg/week of PEG), Jintrolong^®^ is predicted to have minimal-to-no risk under clinical dose too for 3 reasons: 1) The corresponding PEG dose of Jintrolong^®^ is 0.009 µmol/kg/week, lower than 0.011 µmol/kg/week of Skytrofa^®^. 2) The clinical AUC of Jintrolong^®^ (37.229 µg·h/ml) is only as half as Skytrofa^®^ (74 µg·h/ml) in PGHD. 3) The multiple of exposure (MoE) under NOEL of Jintrolong^®^ (4.9×) is higher than Skytrofa^®^ (1.9×), and the MoE under NOAEL of Jintrolong^®^ (120.5×) is higher than Skytrofa^®^ (52.0×).

Based on the quantification and modeling data, the predicted minimum dose to induce vacuolation in monkeys receiving a 1-year treatment is 6.45-fold higher than the clinical dose of 0.2 mg/kg/week Jintrolong^®^. This finding is quite similar to that of Skytrofa. Systemic PEG concentrations at steady state (15 μg/ml) in children with GHD administered Skytrofa were reported to be 7-fold below the PEG exposure vacuolation threshold (100 μg/ml). Based on the data from Skytrofa. the PEG reaches steady state concentration in monkeys with around 1.9 months in blood and 9.6 months in CP. The PEG reaches steady state concentration in PGHD at around 2.9 months in blood and 16 months in CP. Theoretically, the risk of vacuolation for longer administration of PEG-conjugated drugs after a steady state in CP is limited. The steady state PEG concentration of Jintrolong is unavailable, but model simulation data show that the clinical dose is around 6.45 folds lower than the vacuolation threshold as compared with PK data in monkeys. Longer-term administration of Jintrolong under a clinical dose is less likely to increase the vacuolation risk.

Taken together, the total 209 weeks (52-week Jintrolong^®^ administration and 157-week recovery period) nonclinical toxicity study in cynomolgus monkeys combined with 3 years of clinical trial safety data and modeling data represents minimal-to-no risk to GHD children of long-term use of Jintrolong^®^ under clinical dose 0.2 mg/kg/week based on the following consideration:

1) PEG related vacuolation and accumulation of CP epithelium were not observed histologically in cynomolgus monkeys after 52-week administration at 0.3 mg/kg/week (NOEL) with an exposure 4.9-fold higher than the clinical dose, while the NOAEL (3.0 mg/kg/week) exhibits large safety margins of 120.5-fold and 136.1-fold than the clinic dose (0.2 mg/kg/week) based on the AUC and C_max_, respectively.

2) The presence of PEG and vacuolation observed in the CP was not associated with histological evidence of structural changes to tissue architecture, degeneration, necrosis, or inflammation, and no clinical signs of neurotoxicity were observed (e.g., tremors, convulsions, reactivity to handling or unusual behavior).

3) The presence of vacuolation in the CP was not associated with CP cell viability and function, e.g., cell proliferation, apoptosis, CSF components, or glucose metabolism.

4) PEG was not detected in CSF by ELISA above the lower limit of quantification (LLOQ) of 8 ng/mL except for several samples with mixed blood during the CSF collection process in cynomolgus monkeys. PEG was not detected in the brain parenchyma by IHC either. The toxicology results with Jintrolong^®^ appear consistent with the view of the Society of Toxicologic Pathology (STP) Working Group publication (41) and FDA reviewed Lonapegsomatropin (ACP-011) non-clinical document (57).

5) No significant difference in the incidence of CNS-related ARs in pediatric patients treated with 0.2 mg/kg/week of Jintrolong^®^ (0.009 µmol PEG-GH/kg/week) and Jintropin (GH) further supports the long-term use of Jintrolong^®^ in clinical.

## Data Availability Statement

The datasets presented in this study can be found in online repositories. The names of the repository/repositories and accession number(s) can be found in the article/**Supplementary Material**.

## Ethics Statement

The studies involving human participants were reviewed and approved by the Chinese State Food and Drug Administration. Written informed consent to participate in this study was provided by the participants’ legal guardian/next of kin. The animal study was reviewed and approved by the Association for Assessment and Accreditation of Laboratory Animal Care International (AAALAC). Written informed consent was obtained from the minor(s)’ legal guardian/next of kin for the publication of any potentially identifiable images or data included in this article.

## Author Contributions

All authors listed have made a substantial, direct, and intellectual contribution to the work and approved it for publication.

## Funding

The nonclinical research is funded by the Major Projects of Hubei Province (2020AEA009) and the clinical studies were sponsored by GeneScience Pharmaceuticals Co., Ltd (Changchun, China). The funder was not involved in the study design, collection, analysis, interpretation of data, the writing of this article or the decision to submit it for publication.

## Conflict of Interest

JZ, QZ, XLi, TL, LJ and EZ are employees of GeneScience Pharmaceuticals Co., Ltd. CW, CZ, YZ and JY are employees by JOINN Laboratories Inc.

The remaining authors declare that the research was conducted in the absence of any commercial or financial relationships that could be construed as a potential conflict of interest.

## Publisher’s Note

All claims expressed in this article are solely those of the authors and do not necessarily represent those of their affiliated organizations, or those of the publisher, the editors and the reviewers. Any product that may be evaluated in this article, or claim that may be made by its manufacturer, is not guaranteed or endorsed by the publisher.

## Supplementary Material

Supplementary Figure 1 H&E staining of different tissues of cynomolgus monkeys with 52-week administration of Jintrolong. **(A)**. Kidney of excipient control group with 52-week administration. **(B)**. Lung of excipient control group with 52-week administration. **(C)**. Heart of excipient control group with 52-week administration. **(D)**. Spleen of excipient control group with 52-week administration. **(E)**. Liver of excipient control group with 52-week administration. **(F–J)**. Kidney, lung, heart, spleen and liver of Jintrolong group with 0.3mg/kg/week administration for 52 weeks. **(K–O)**. Kidney, lung, heart, spleen and liver of Jintrolong group with 1mg/kg/week administration for 52 weeks. **(P–T)**. Kidney, lung, heart, spleen and liver of Jintrolong group with 3mg/kg/week administration for 52 weeks. The images of kidney and heart were taken under 20x, the images of lung, spleen and liver were taken under 10x. The kidney and heart were shot by 20x to see the glomerulus or cardiomyocytes more clearly, while the liver, spleen, and lung shots had a larger field of view because the liver had to take the center of the lobules and the portal area, and the spleen had to be shot to see the red and white pulp, the lungs must be photographed of the alveoli and bronchi, so the magnification should not be large.

Supplementary Figure 2 H&E staining of macrophages in cynomolgus monkeys with 52-week administration of Jintrolong. **(A)**. Lung of Jintrolong group with 3mg/kg/week administration for 52 weeks, arrows indicate the macrophages in lung. **(B)**. Liver of Jintrolong group with 3mg/kg/week administration for 52 weeks, arrows indicate the Kupffer cells in liver. **(C)**. Spleen of Jintrolong group with 3mg/kg/week administration for 52 weeks, arrows indicate the macrophages in spleen. **(D)**. Brain of Jintrolong group with 3mg/kg/week administration for 52 weeks, arrows indicate the microglias in brain.

Supplementary Figure 3 Subcellular imaging of cynomolgus monkeys CP epithelium with 52-week administration of Jintrolong and after 104-/157- week recovery. **(A)**. Representative image for excipient control group. **(B)**. Area in monkey #7883(3 mg/kg/week Jintrolong group with 52 weeks administration) show minor subcellular structure change. **(C)**. 3 mg/kg/week Jintrolong group with 52 weeks administration and 104 weeks recovery. **(D)**. 3 mg/kg/week Jintrolong group with 52 weeks administration and 157 weeks recovery. Left arrow with TJ in **Figure S3B** indicate intact tight junction. Arrow with ERD in **Figure S3B** indicate rough endoplasmic reticulum dilation. The lower arrow with MS in **Figure S3B** indicate minor mitochondrial swelling with bigger size, lighter color and less cristae. Arrow with LMV in **Figure S3B** indicate less microvilli with enlarged terminal vesicles.
