# Supplementary figures and images for: PEGylated Recombinant Human Growth Hormone Jintrolong® Exhibits Good Long-Term Safety in Cynomolgus Monkeys and Human Pediatric Growth Hormone Deficiency Patients
Source: Front Endocrinol (Lausanne). 2022 Jul 15;13:821588. doi: 10.3389/fendo.2022.821588 (PMC9336684; doi:10.3389/fendo.2022.821588)

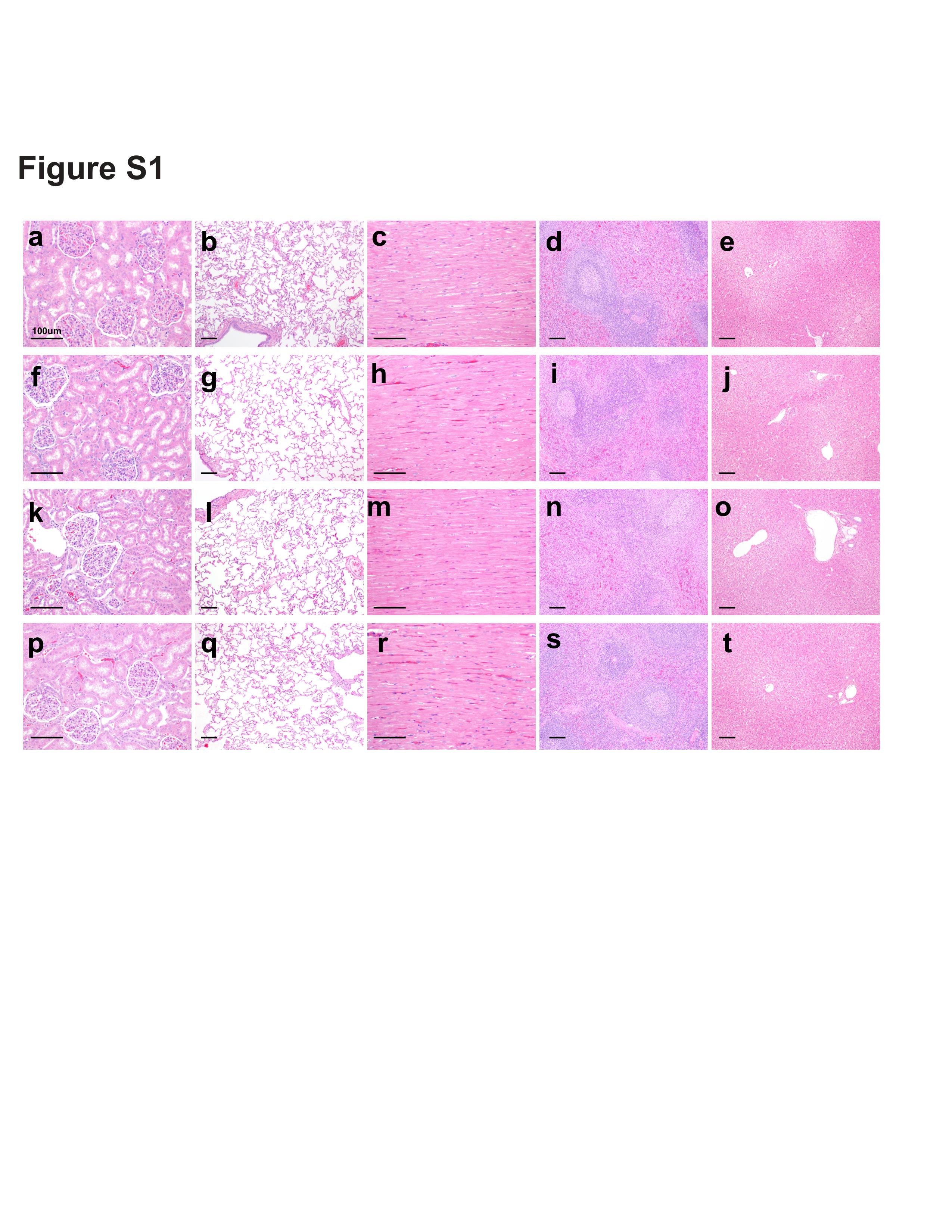

Supplement: Supplementary Figure 1 — H&E staining of different tissues of cynomolgus monkeys with 52-week administration of Jintrolong. (A). Kidney of excipient control group with 52-week administration. (B). Lung of excipient control group with 52-week administration. (C). Heart of excipient control group with 52-week administration. (D). Spleen of excipient control group with 52-week administration. (E). Liver of excipient control group with 52-week administration. (F–J). Kidney, lung, heart, spleen and liver of Jintrolong group with 0.3mg/kg/week administration for 52 weeks. (K–O). Kidney, lung, heart, spleen and liver of Jintrolong group with 1mg/kg/week administration for 52 weeks. (P–T). Kidney, lung, heart, spleen and liver of Jintrolong group with 3mg/kg/week administration for 52 weeks. The images of kidney and heart were taken under 20x, the images of lung, spleen and liver were taken under 10x. The kidney and heart were shot by 20x to see the glomerulus or cardiomyocytes more clearly, while the liver, spleen, and lung shots had a larger field of view because the liver had to take the center of the lobules and the portal area, and the spleen had to be shot to see the red and white pulp, the lungs must be photographed of the alveoli and bronchi, so the magnification should not be large. [file Image_1.jpeg]

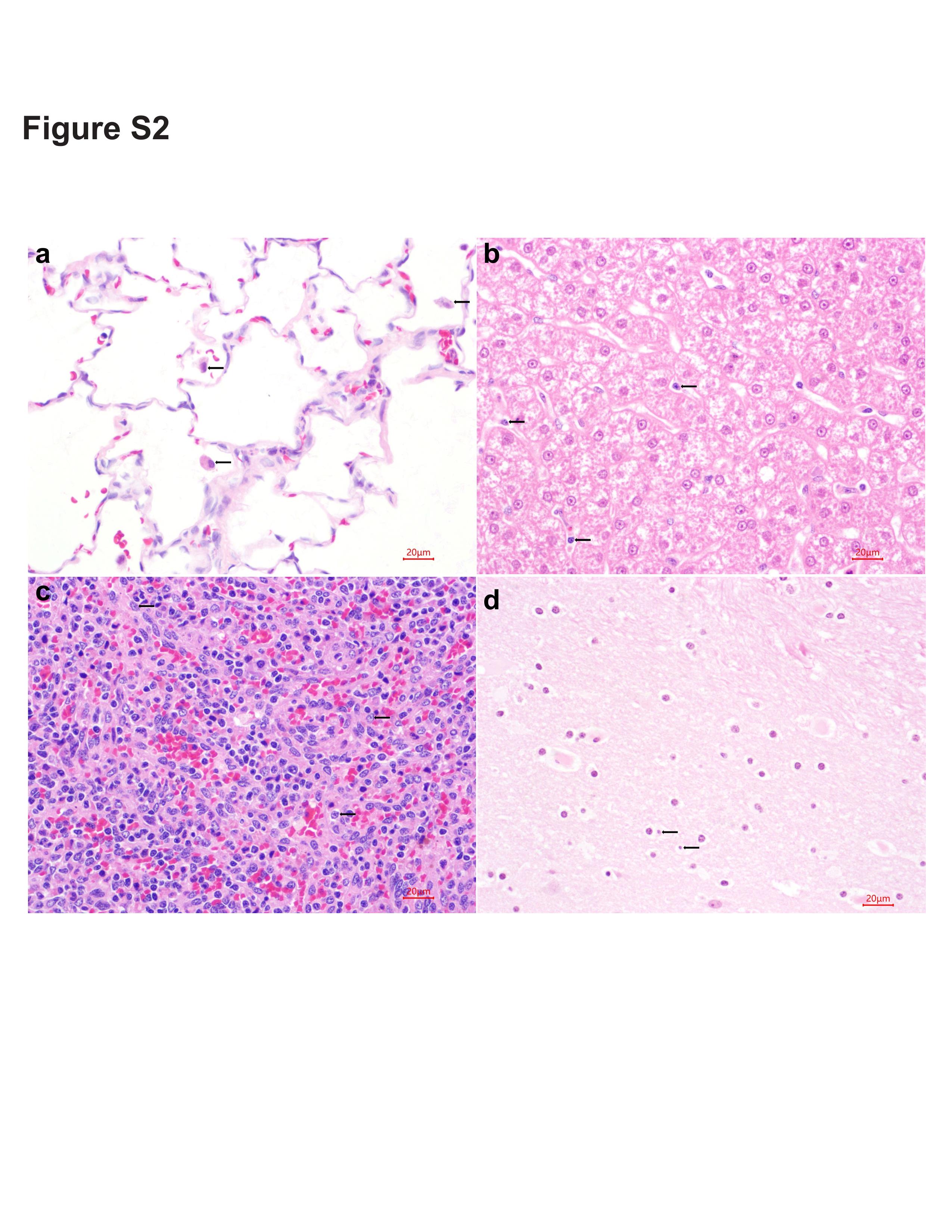

Supplement: Supplementary Figure 2 — H&E staining of macrophages in cynomolgus monkeys with 52-week administration of Jintrolong. (A). Lung of Jintrolong group with 3mg/kg/week administration for 52 weeks, arrows indicate the macrophages in lung. (B). Liver of Jintrolong group with 3mg/kg/week administration for 52 weeks, arrows indicate the Kupffer cells in liver. (C). Spleen of Jintrolong group with 3mg/kg/week administration for 52 weeks, arrows indicate the macrophages in spleen. (D). Brain of Jintrolong group with 3mg/kg/week administration for 52 weeks, arrows indicate the microglias in brain. [file Image_2.jpeg]

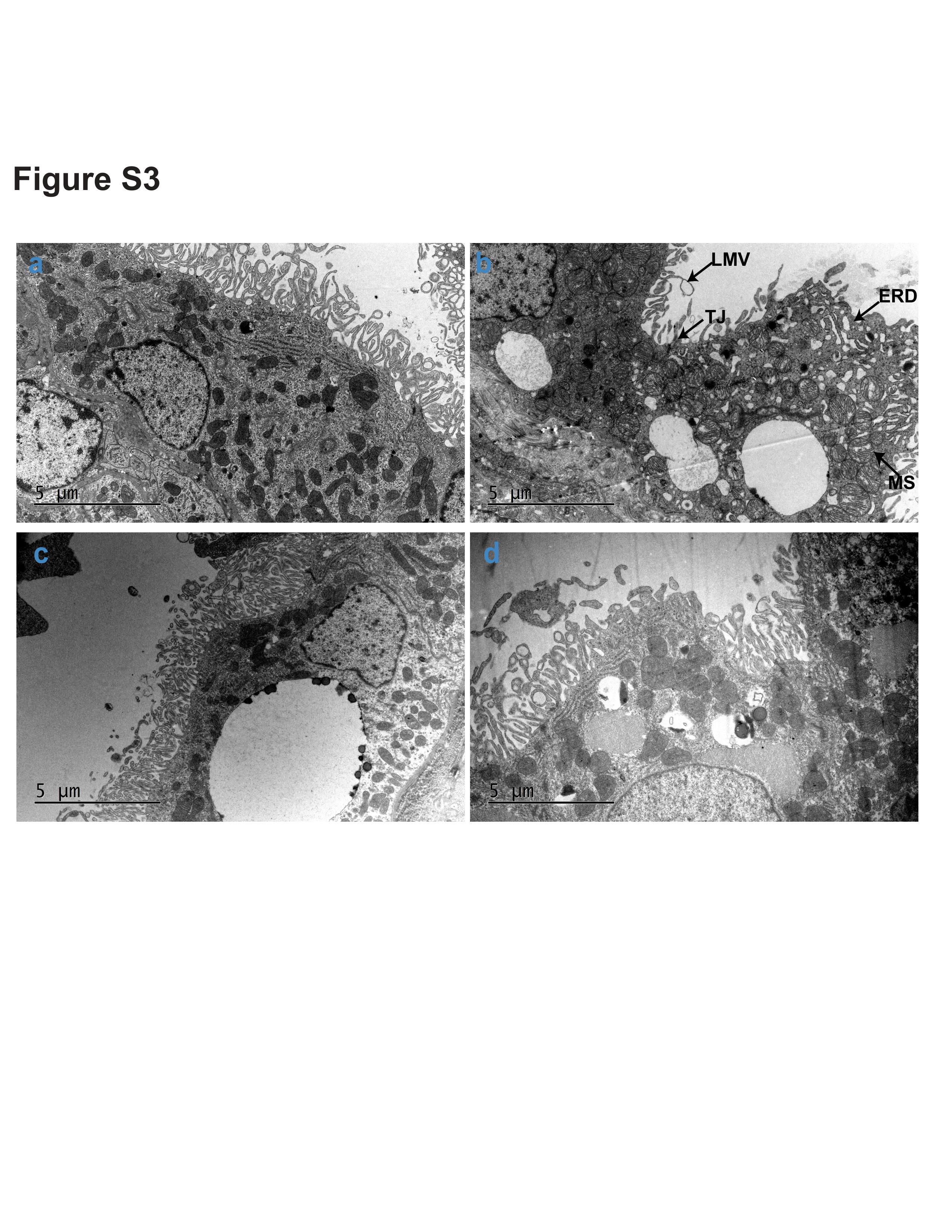

Supplement: Supplementary Figure 3 — Subcellular imaging of cynomolgus monkeys CP epithelium with 52-week administration of Jintrolong and after 104-/157- week recovery. (A). Representative image for excipient control group. (B). Area in monkey #7883(3 mg/kg/week Jintrolong group with 52 weeks administration) show minor subcellular structure change. (C). 3 mg/kg/week Jintrolong group with 52 weeks administration and 104 weeks recovery. (D). 3 mg/kg/week Jintrolong group with 52 weeks administration and 157 weeks recovery. Left arrow with TJ in Figure S3B indicate intact tight junction. Arrow with ERD in Figure S3B indicate rough endoplasmic reticulum dilation. The lower arrow with MS in Figure S3B indicate minor mitochondrial swelling with bigger size, lighter color and less cristae. Arrow with LMV in Figure S3B indicate less microvilli with enlarged terminal vesicles. [file Image_3.jpeg]
