# Supplementary material for: PEGylated Recombinant Human Growth Hormone Jintrolong® Exhibits Good Long-Term Safety in Cynomolgus Monkeys and Human Pediatric Growth Hormone Deficiency Patients
Source: Front Endocrinol (Lausanne). 2022 Jul 15;13:821588. doi: 10.3389/fendo.2022.821588 (PMC9336684; doi:10.3389/fendo.2022.821588)
Supplement: Supplementary file 4 [file Table_1.pdf]

Table S1. Observations and examinations for general healthy assessments

Data collection	Test article-related changes
Mortality and Moribundity	N
Clinical observations	N
Body weights	N
Body temperature	N
Electrocardiogram	N
Blood pressure	N
Ophthalmoscopic Examinations	N
Hematology	N
Coagulation	N
Clinical chemistry	N
Urinalysis	N
Lymphocyte Subpopulation	N
Serum Hormone	N
